# Supplementary material for: Genome-wide association studies and Mendelian randomization analyses for leisure sedentary behaviours
Source: Nat Commun. 2020 Apr 21;11:1770. doi: 10.1038/s41467-020-15553-w (PMC7174427; doi:10.1038/s41467-020-15553-w)
Supplement: Supplementary file 1 — Supplementary Information [file 41467_2020_15553_MOESM1_ESM.pdf]

Genome-wide association studies and Mendelian randomization analyses for leisure sedentary behaviours

Van de Vegte *et al*.

Supplementary Information

Supplementary Figures

Supplementary Figure 1: Manhattan plot shows the results for the genome-wide associations with leisure television watching among individuals of European origin. Loci reaching genome-wide significance ($P< 1 \times 10^{-8}$) are colored red.

Supplementary Figure 2: Manhattan plot shows the results for the genome-wide associations with leisure computer use among individuals of European origin. Loci reaching genome-wide significance ($P<1 \times 10^{-8}$) are colored red.

Supplementary Figure 3: Manhattan plot shows the results for the genome-wide associations with driving among individuals of European origin. Loci reaching genome-wide significance ($P<1 \times 10^{-8}$) are colored red.

Supplementary Figure 4: Scatter plot of leisure television watching genetic variants and coronary artery disease. The variants' effects on leisure television watching are displayed on the X-axis, the variants' effects on coronary artery disease on the Y-axis.

Supplementary Figure 5: Scatter plot of leisure computer use genetic variants and coronary artery disease. The variants' effects on leisure computer use are displayed on the X-axis, the variants' effects on coronary artery disease on the Y-axis.

Supplementary Figure 6: Scatter plot of driving genetic variants and coronary artery disease. The variants' effects on driving are displayed on the X-axis, the variants' effects on coronary artery disease on the Y-axis.

Supplementary Figure 7: Forest plot of leisure television watching genetic variants associations with coronary artery disease. The Mendelian Randomization effect size of television watching on coronary artery disease product are displayed on the X-axis. The different genetic variants for television watching are listed on the Y-axis.

Supplementary Figure 8: Forest plot of leisure computer use genetic variants associations with coronary artery disease. The Mendelian randomization effect size of television watching on coronary artery disease product are displayed on the X-axis. The different genetic variants for television watching are listed on the Y-axis.

Supplementary Figure 9: Forest plot of driving genetic variants associations with coronary artery disease. The Mendelian randomization effect size of television watching on coronary artery disease product are displayed on the X-axis. The different genetic variants for television watching are listed on the Y-axis.

Supplementary Figure 10: quantile–quantile (QQ) plot for leisure television watching.

Supplementary Figure 11: quantile–quantile (QQ) plot for leisure computer use.

Supplementary Figure 12: quantile–quantile (QQ) plot for driving.

Supplementary Tables

Supplementary Table 1: Correlations among sedentary behaviour traits.

Supplementary Table 2: Association of sedentary behaviour traits with possible confounding factors in the observational analyses

Supplementary Table 3: Association of sedentary behaviour traits with new onset coronary artery disease incidence in the observational analyses.

Supplementary Table 4: Results of the two-sample Mendelian randomization analyses of sedentary traits on coronary artery disease, including the multivariable MR with education.

Supplementary Table 5: Heterogeneity (I^2, Cochran's Q, Rucker's Q and Q-Q'), pleiotropy (MR-Egger intercept) and weak instrument statistics in the MR-Egger analyses (I^2_{GX}) in the examined associations between sedentary behaviours and coronary artery disease.

Supplementary Table 6: SNPs excluded from the outlier corrected MR-PRESSO analyses between sedentary behaviours and coronary artery disease.

Supplementary Table 7: Potentially pleiotropic SNPs in the Mendelian randomization between sedentary behaviours coronary artery disease.

Supplementary Table 8: Results from TwoSample Mendelian randomization analysis between education and sedentary behaviours traits.

Supplementary Table 9: Heterogeneity (I^2, Cochran's Q, Rucker's Q and Q-Q'), pleiotropy (MR-Egger intercept) and weak instrument statistics in the MR-Egger analysis (I^2_{GX}) in the examined associations between education and sedentary behaviours.

Supplementary Table 10: SNPs excluded from the outlier corrected MR-PRESSO analyses between education and sedentary behaviours.

Supplementary Table 11: Q_{x1} and Q_{x2} and Q_a in the two-sample multivariable MR between sedentary behaviours, education and CAD.

Supplementary Table 12: Results of the additional two-sample Mendelian randomization analyses between sedentary behaviours and cardiovascular risk factors, and subsequent multivariable Mendelian randomization analyses on CAD.

Supplementary Discussion

Supplementary References

Additional online data

Supplementary Data 1: 193 Novel genome-wide sedentary behaviour SNPs (.xlsx).

Supplementary Data 2: Genetic correlation between sedentary behaviours, and previously performed GWAS's (.xlsx).

Supplementary Data 3: Sedentary behaviour variants associated with previously discovered variants (.xlsx).

Supplementary Data 4: List of coding variants (.xlsx).

Supplementary Data 5: List of functional eQTL genes (.xlsx).

Supplementary Data 6: List of DEPICT genes (.xlsx).

Supplementary Data 7: Results of gene sets discovered by DEPICT for sedentary behaviour and sedentary behaviour traits separately (.xlsx).

Supplementary Data 8: Results of enriched tissue sets discovered by DEPICT for sedentary behaviour and sedentary behaviour traits separately (.xlsx).

Supplementary Data 9: Genetic association estimates for the association between sedentary behaviours and coronary artery disease (.xlsx).

Supplementary Data 10: F-statistics and R^2 of all instruments for the Mendelian randomization analysis between sedentary behaviours and coronary artery disease (.xlsx).

Supplementary Data 11: Inverse variance weighted Mendelian randomization estimates for the association between education and sedentary behaviours (.xlsx).

Supplementary Data 12: Genetic association estimates for the two-sample regression-based multivariable Mendelian randomization analyses estimating the effect of sedentary behaviours on coronary artery disease, adjusted for education (.xlsx).

Supplementary Data 13: Genetic association estimates for the two-sample regression-based multivariable Mendelian randomization analyses estimating the effect of sedentary behaviours on coronary artery disease, adjusted for cardiovascular risk factors. (.xlsx).

Supplementary Figure 1: Manhattan plot leisure television watching

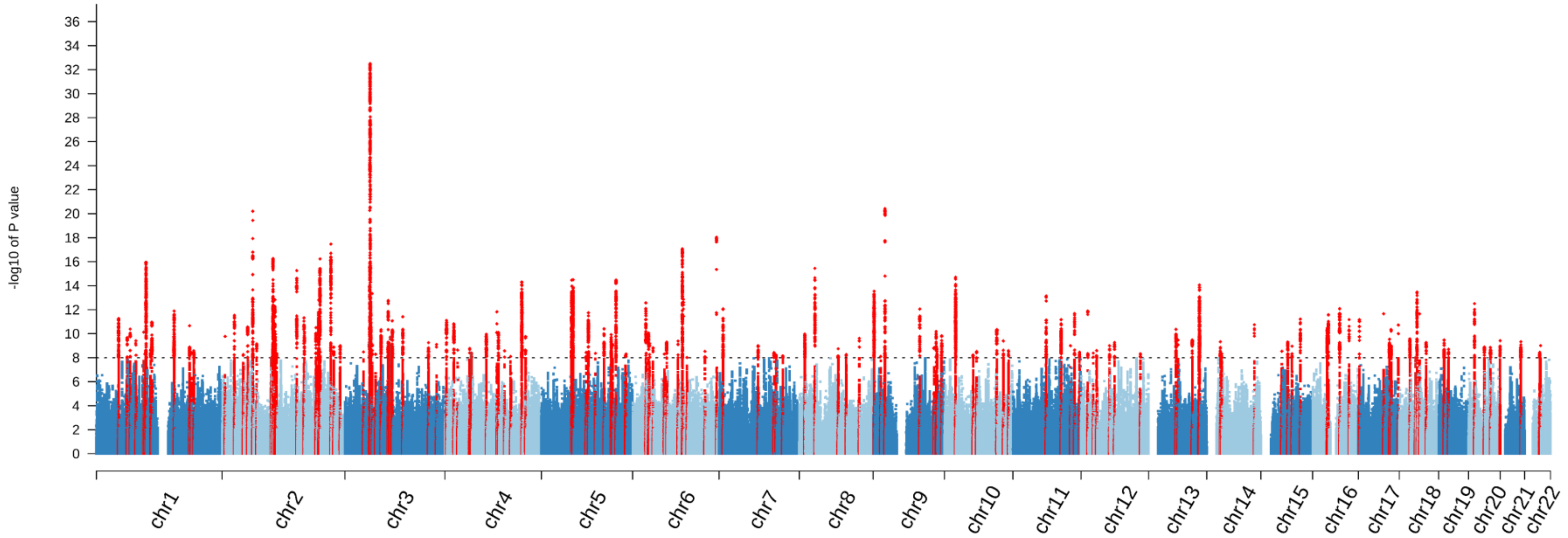

Manhattan plot shows the results for the genome-wide associations with television watching among individuals of European origin. Loci reaching genome-wide significance ($P < 1 \times 10^{-8}$) are colored red. We adopted a stringent (two-sided) genome-wide association threshold in order to in order to account for testing three independent traits.

Supplementary Figure 2: Manhattan plot leisure computer use

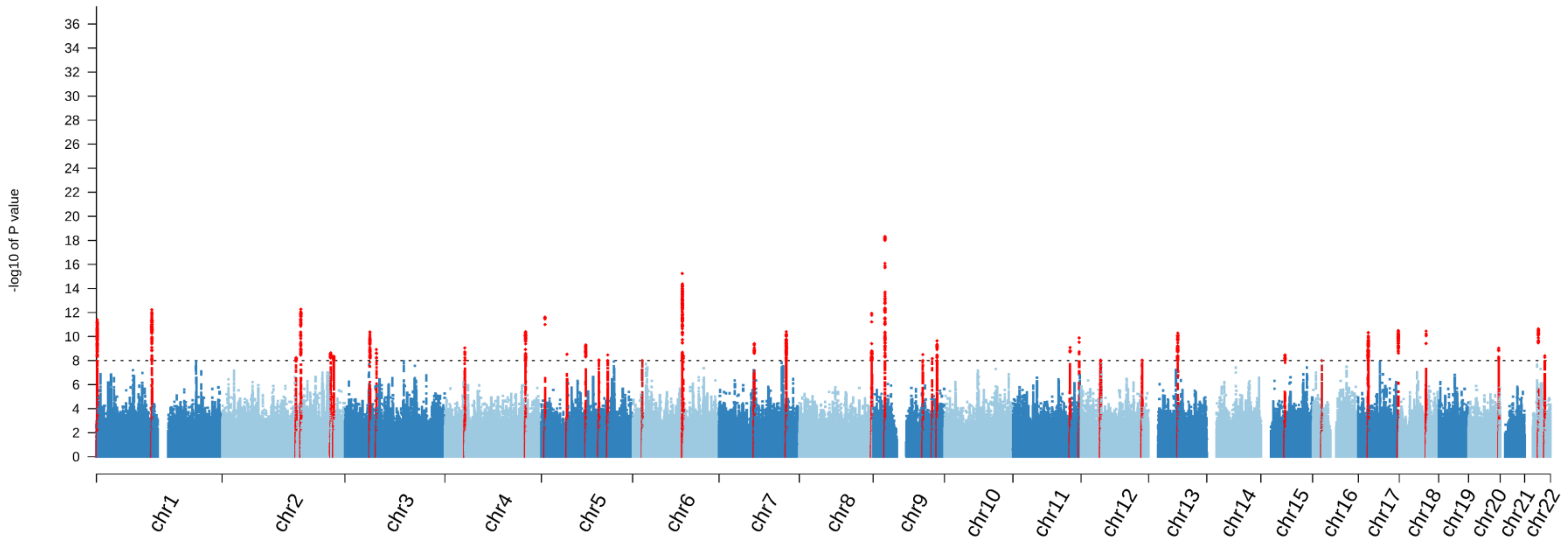

Manhattan plot shows the results for the genome-wide associations with television watching among individuals of European origin. Loci reaching genome-wide significance ($P < 1 \times 10{-}8$) are colored red. We adopted a stringent (two-sided) genome-wide association threshold in order to in order to account for testing three independent traits.

Supplementary Figure 3: Manhattan plot driving

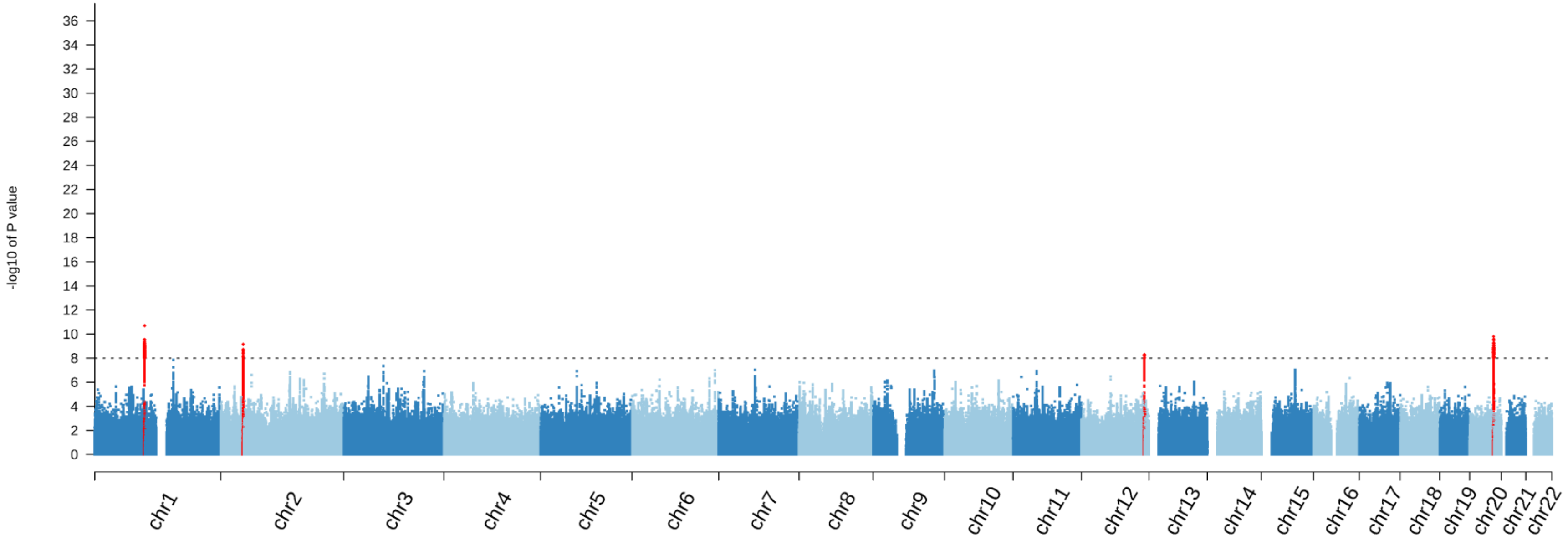

Manhattan plot shows the results for the genome-wide associations with television watching among individuals of European origin. Loci reaching genome-wide significance (P< 1 × 10−8) are colored red. We adopted a stringent (two-sided) genome-wide association threshold in order to in order to account for testing three independent traits.

Supplementary Figure 4: Scatter plot of leisure television watching genetic variants and coronary artery disease

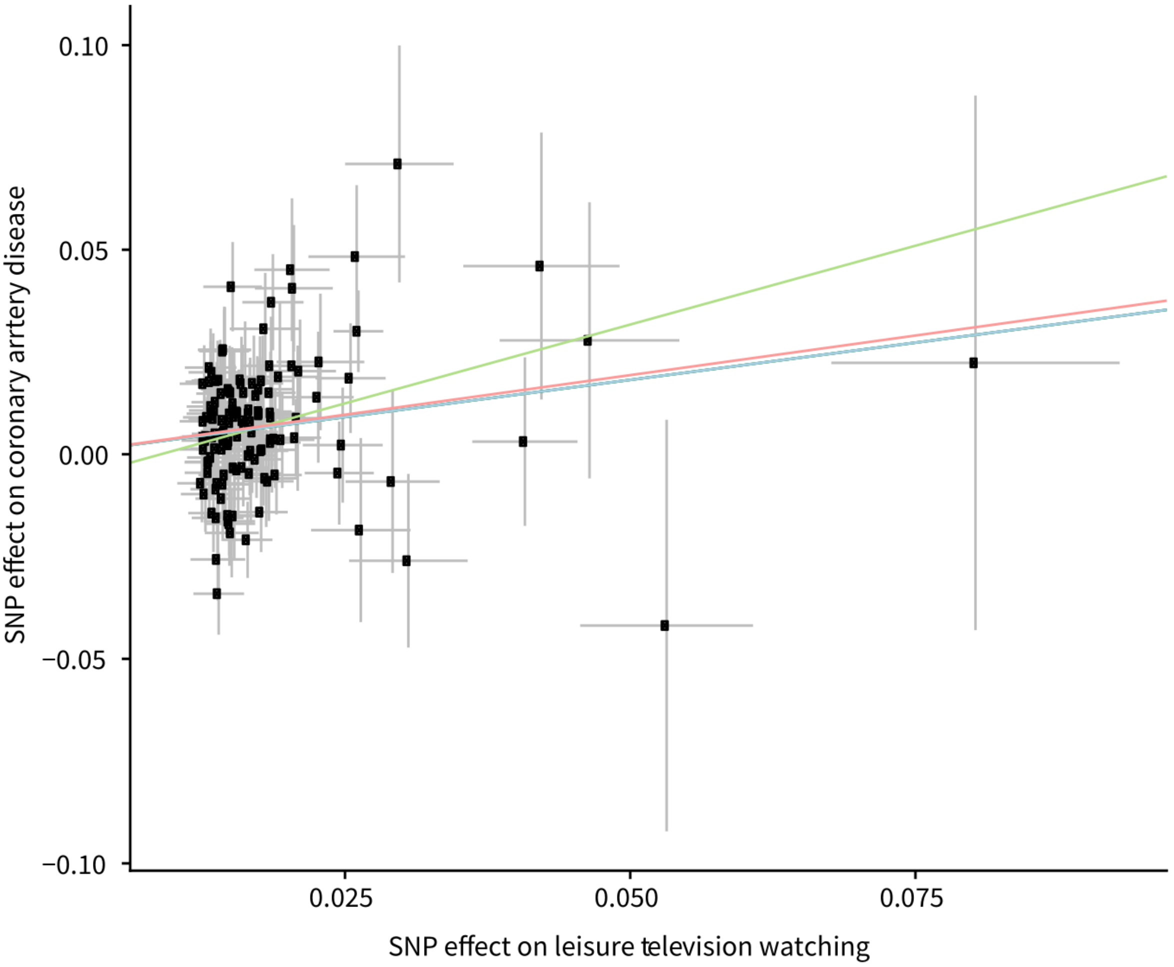

Scatter plot including the MR estimates between leisure television watching and coronary artery disease for the main analyses, in which a threshold of P< 1 × 10^{-8} was used for the selection of variants. The variants' effect size and standard error on leisure television watching are displayed on the X-axis, the variants' effect size and standard error on coronary artery disease on the Y-axis.
The dark blue line is the regression line of the inverse variance weighted fixed effects meta-analysis, the light blue line of the inverse variance weighted random effects meta-analysis, the light green line of the MR-Egger analysis, the dark green line of the weighted median analysis and pink line of the weighted mode analysis.
N=417,555 biologically independent individuals from the UK Biobank were used to obtain effect estimates of the genetic variants associated with sedentary behaviours. N=184,305 (60,801 cases, 123,504 controls) individuals were used to obtain effect estimates of the genetic variants associated with CAD. A total of 127 single SNP associations are shown.
SNP denotes single nucleotide polymorphism, MR denotes Mendelian randomization.

Supplementary Figure 5: Scatter plot of leisure computer use genetic variants and coronary artery disease

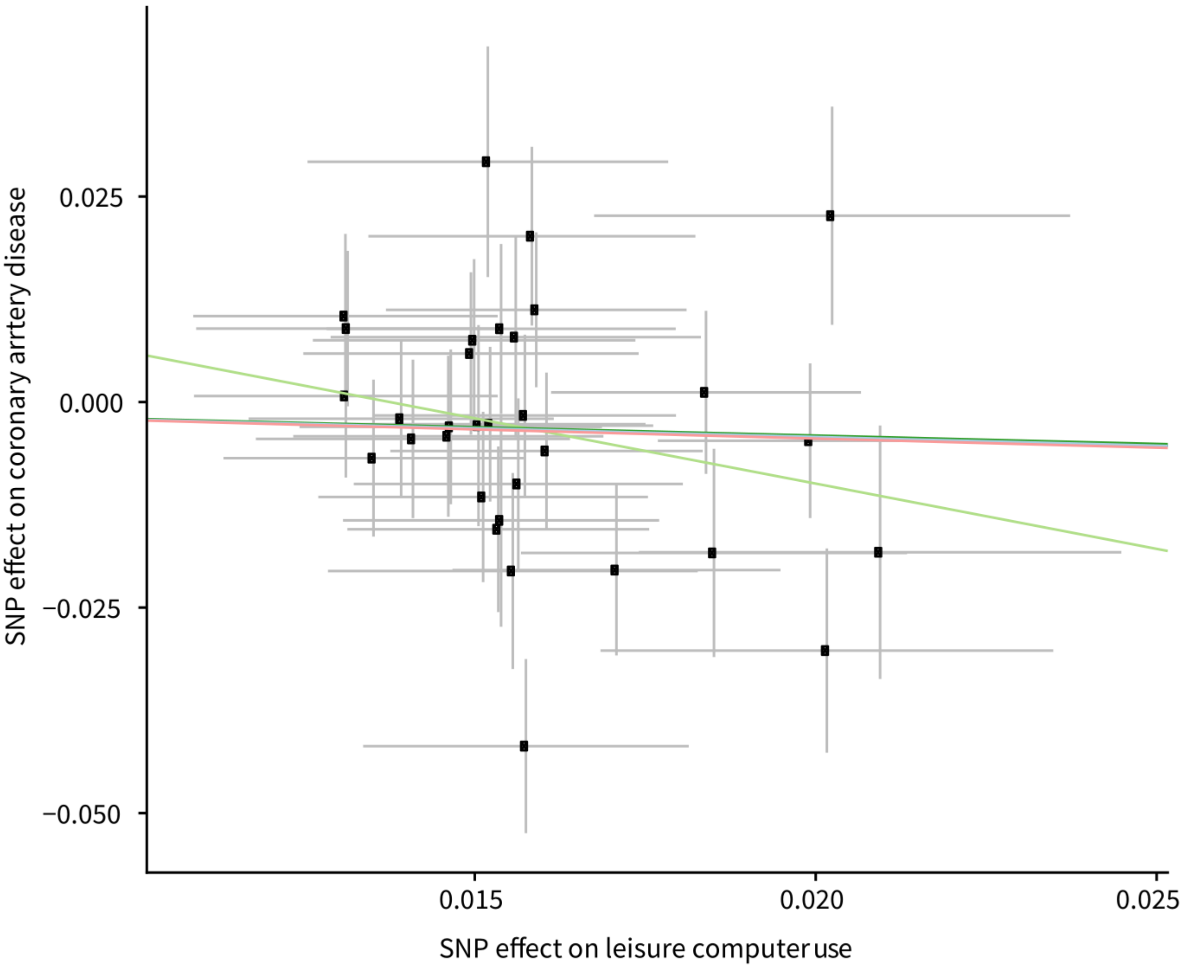

Scatter plot including the MR estimates between leisure computer use and coronary artery disease for the main analyses, in which a threshold of $P< 1 \times 10^{-8}$ was used for the selection of variants. The variants' effect size and standard error on leisure computer use are displayed on the X-axis, the variants' effect size and standard error on coronary artery disease on the Y-axis. The dark blue line is the regression line of the inverse variance weighted fixed effects meta-analysis, the light blue line of the inverse variance weighted random effects meta-analysis, the light green line of the MR-Egger analysis, the dark green line of the weighted median analysis and pink line of the weighted mode analysis.
N=414,927 biologically independent individuals from the UK Biobank were used to obtain effect estimates of the genetic variants associated with computer use. N=184,305 (60,801 cases, 123,504 controls) individuals were used to obtain effect estimates of the genetic variants associated with CAD. A total of 32 single SNP associations are shown.
SNP denotes single nucleotide polymorphism, MR denotes Mendelian randomization.

Supplementary Figure 6: Scatter plot of driving genetic variants and coronary artery disease

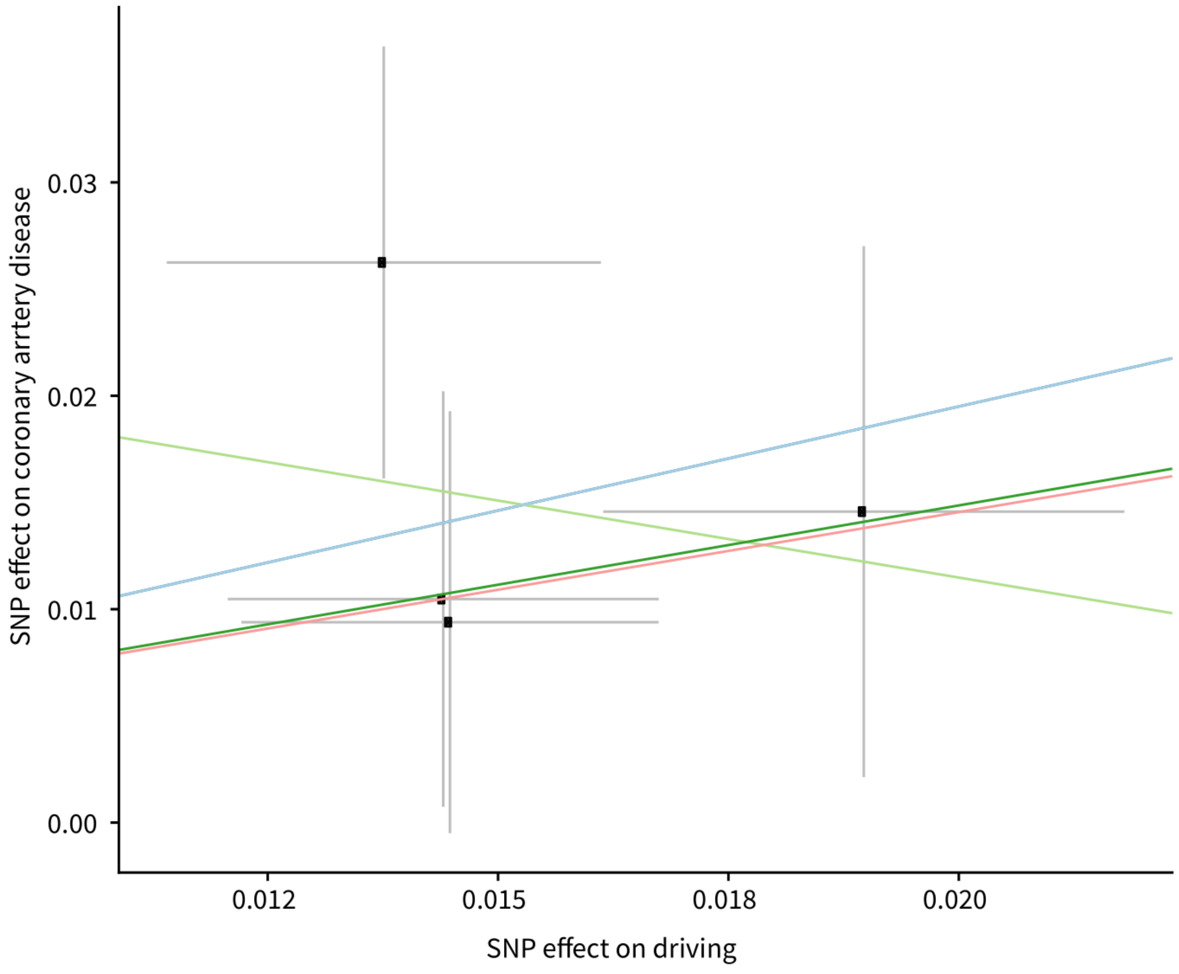

Scatter plot including the MR estimates between driving and coronary artery disease for the main analyses, in which a threshold of P< 1 × 10^{-8} was used for the selection of variants. The variants' effect size and standard error on driving are displayed on the X-axis, the variants' effect size and standard error on coronary artery disease on the Y-axis.
The dark blue line is the regression line of the inverse variance weighted fixed effects meta-analysis, the light blue line of the inverse variance weighted random effects meta-analysis, the light green line of the MR-Egger analysis, the dark green line of the weighted median analysis and pink line of the weighted mode analysis.
N=422,271 biologically independent individuals from the UK Biobank were used to obtain effect estimates of the genetic variants associated with driving. N=184,305 (60,801 cases, 123,504 controls) individuals were used to obtain effect estimates of the genetic variants associated with CAD. A total of 4 single SNP associations are shown.
SNP denotes single nucleotide polymorphism, MR denotes Mendelian randomization.

Supplementary Figure 7: Forest plot of leisure television watching genetic variants associations with coronary artery disease.

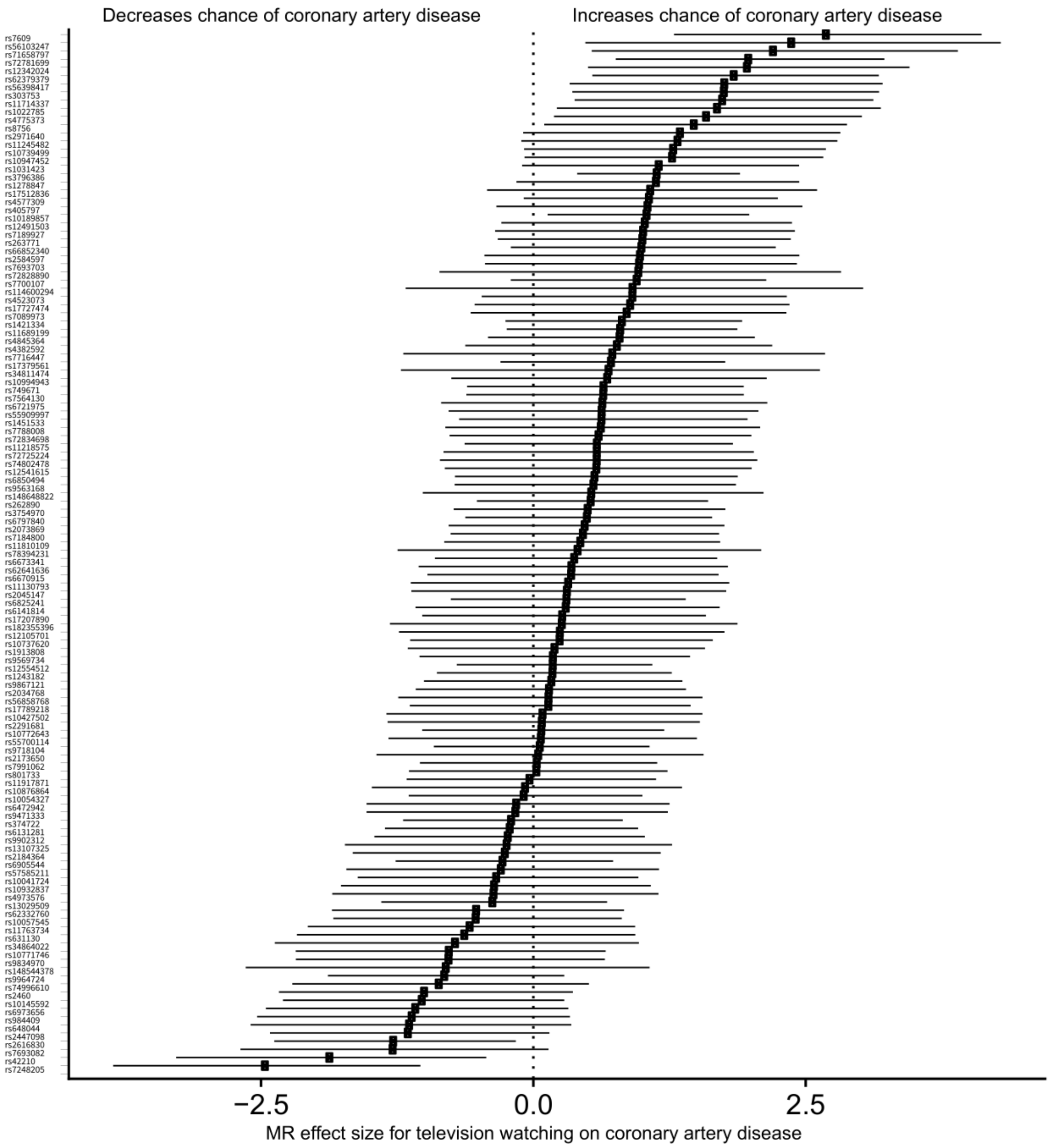

The Mendelian Randomization effect size and standard error of television watching on coronary artery disease product are displayed on the X-axis. The different genetic variants for television watching are listed on the Y-axis.

N=417,555 biologically independent individuals from the UK Biobank were used to obtain effect estimates of the genetic variants associated with sedentary behaviours. N=184,305 (60,801 cases, 123,504 controls) individuals were used to obtain effect estimates of the genetic variants associated with CAD. A total of 127 single SNP associations are shown.

Supplementary Figure 8: Forest plot of leisure computer use watching genetic variants associations with coronary artery disease.

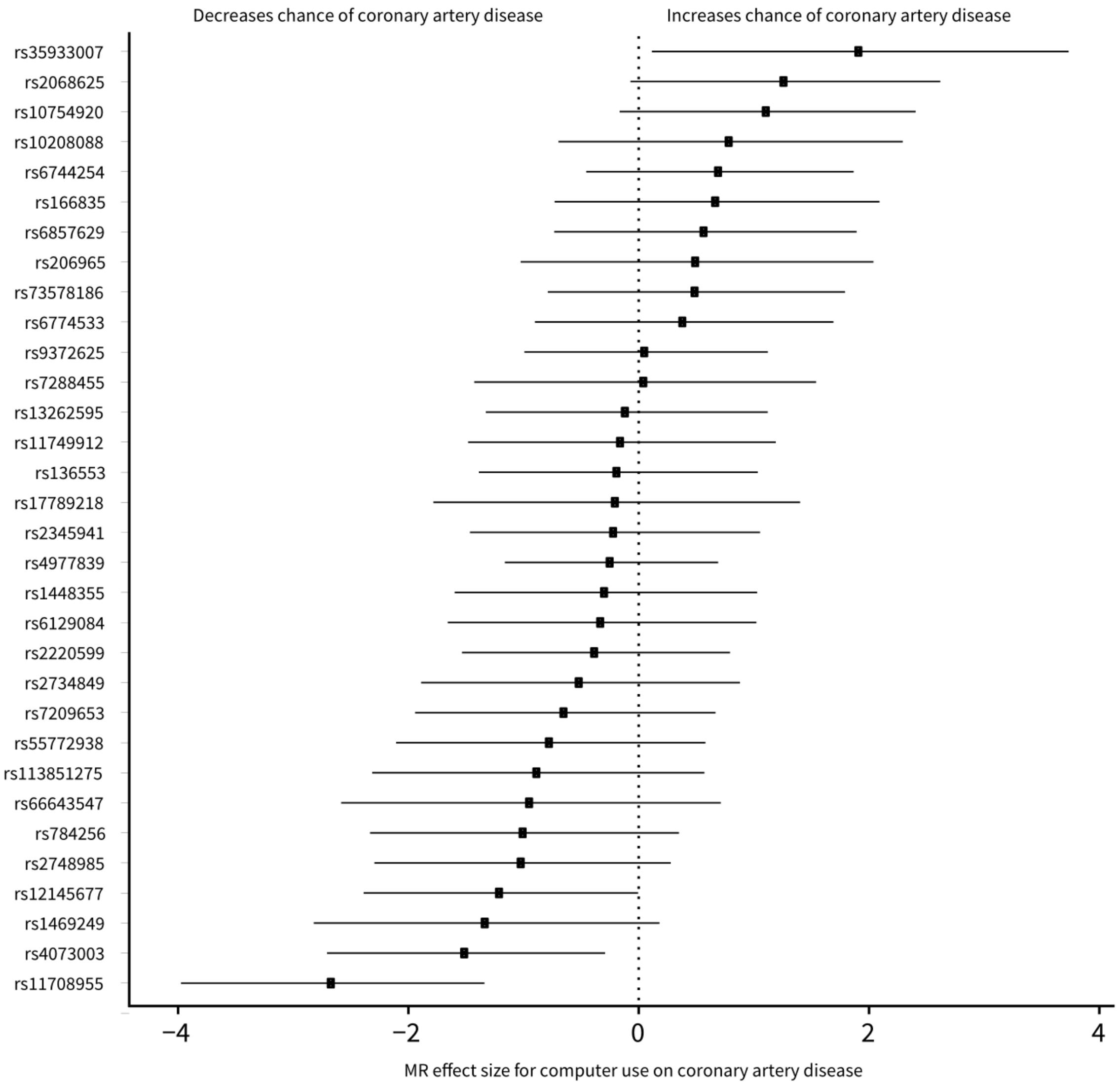

The Mendelian Randomization effect size and standard error of computer use on coronary artery disease product are displayed on the X-axis. The different genetic variants for computer use are listed on the Y-axis. N=414,927 biologically independent individuals from the UK Biobank were used to obtain effect estimates of the genetic variants associated with computer use. N=184,305 (60,801 cases, 123,504 controls) individuals were used to obtain effect estimates of the genetic variants associated with CAD. A total of 32 single SNP associations are shown.

Supplementary Figure 9: Forest plot of driving genetic variants associations with coronary artery disease.

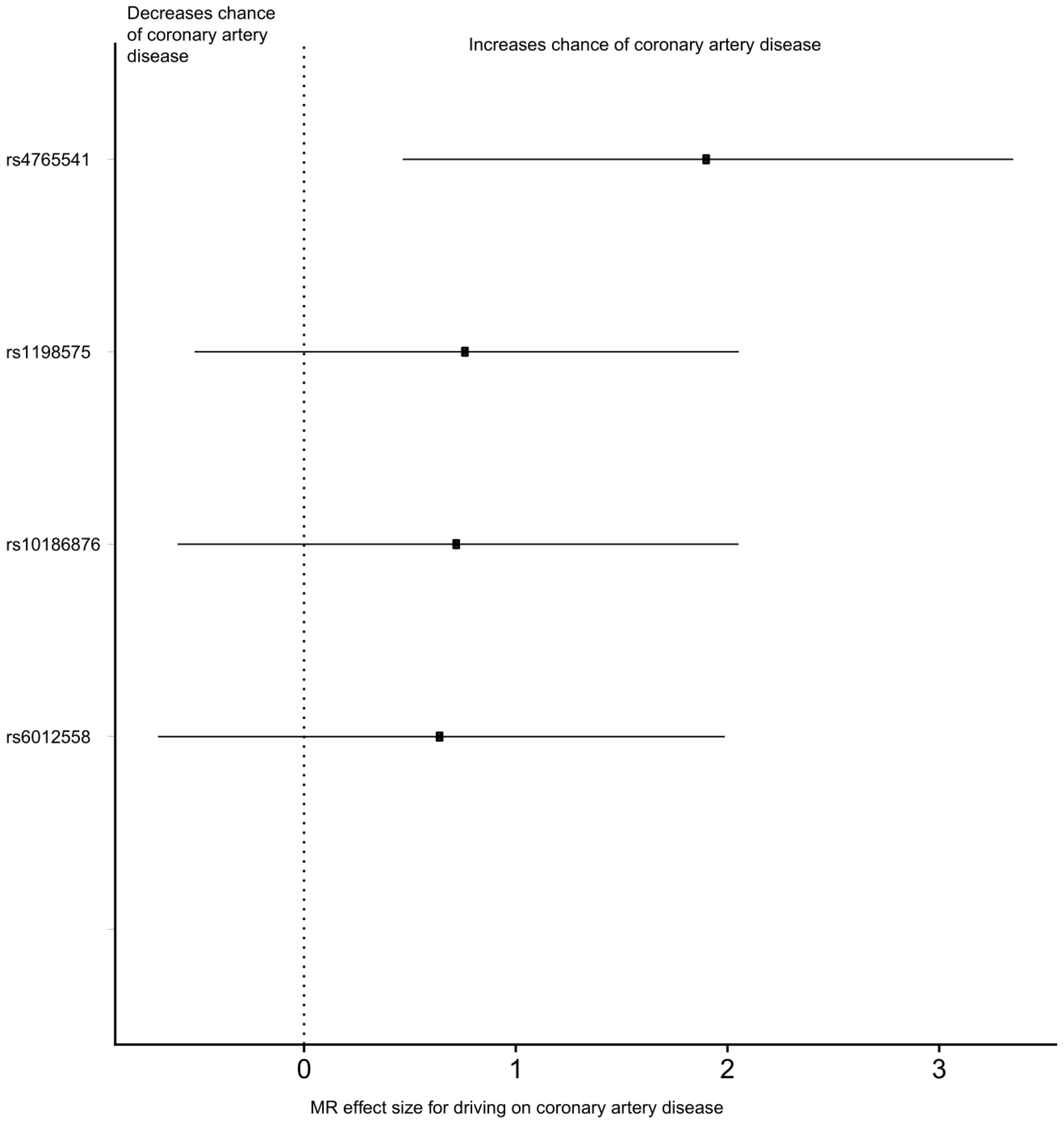

The Mendelian Randomization effect size and standard error of driving on coronary artery disease product are displayed on the X-axis. The different genetic variants for driving are listed on the Y-axis.
N=422,271 biologically independent individuals from the UK Biobank were used to obtain effect estimates of the genetic variants associated with driving. N=184,305 (60,801 cases, 123,504 controls) individuals were used to obtain effect estimates of the genetic variants associated with CAD. A total of 4 single SNP associations are shown.

Supplementary Figure 10: quantile–quantile (QQ) plot for leisure television watching.

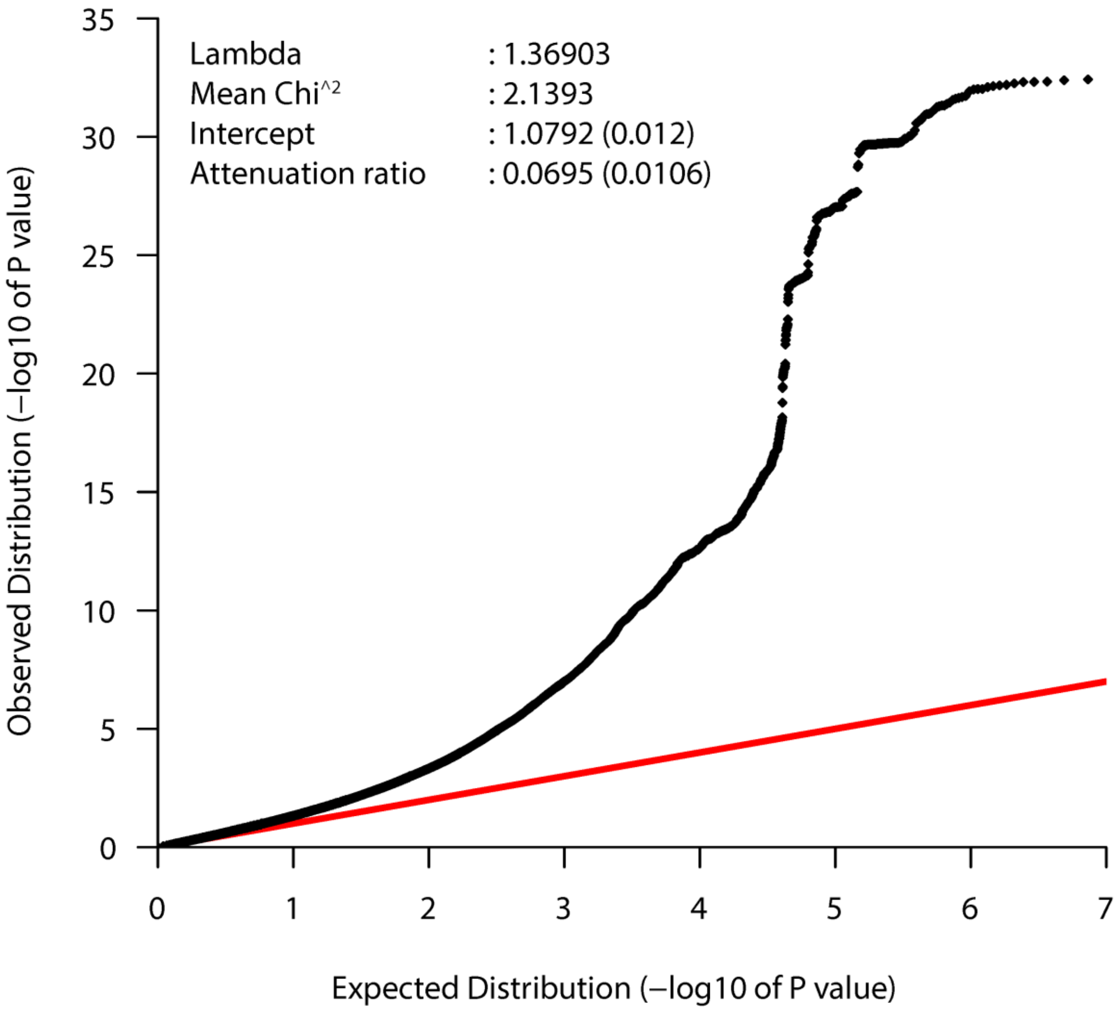

Quantile–quantile (QQ) plot for the GWAS of leisure television watching. The genomic intercept indicated a possibility of population stratification. However, attenuation ratio statistic indicated polygenicity, not population stratification, to be the main driver of the observed inflation of test statistics for television watching.
The X-axis shows the expected distribution in –log$_{10}$(P-value). The Y-axis the observed distribution in –log$_{10}$(P-value). The red line follows expected P-values from a theoretical χ^2-distribution, whereas the black line follows the observed P-values in the current GWAS.

Supplementary Figure 11: quantile–quantile (QQ) plot for leisure computer use.

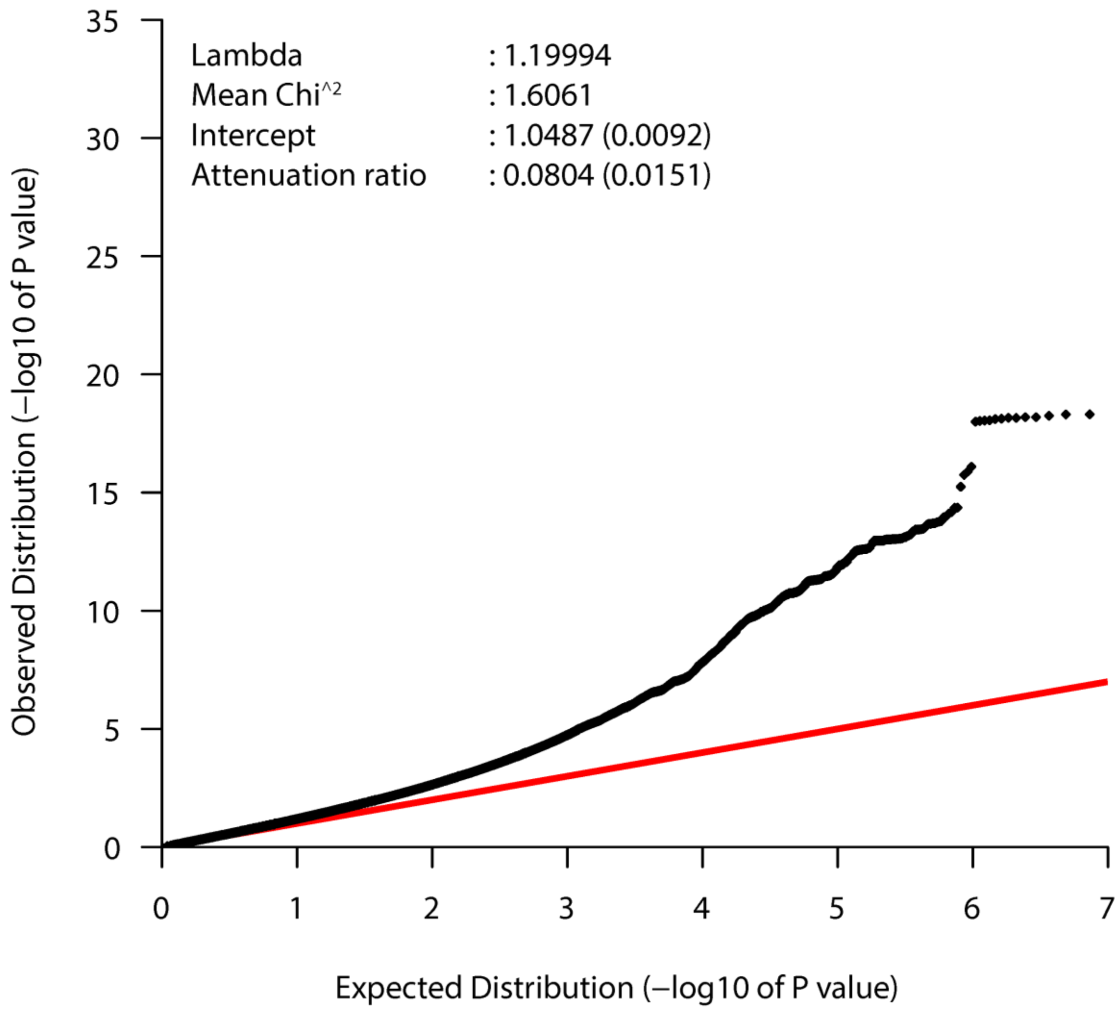

Quantile–quantile (QQ) plot for the GWAS of leisure computer use. LD score regression intercepts showed no genomic inflation due to non-polygenic signals for computer use.
The X-axis shows the expected distribution in –log_{10}(P-value). The Y-axis the observed distribution in –log_{10}(P-value). The red line follows expected P-values from a theoretical χ^2-distribution, whereas the black line follows the observed P-values in the current GWAS.

Supplementary Figure 12: quantile–quantile (QQ) plot for driving.

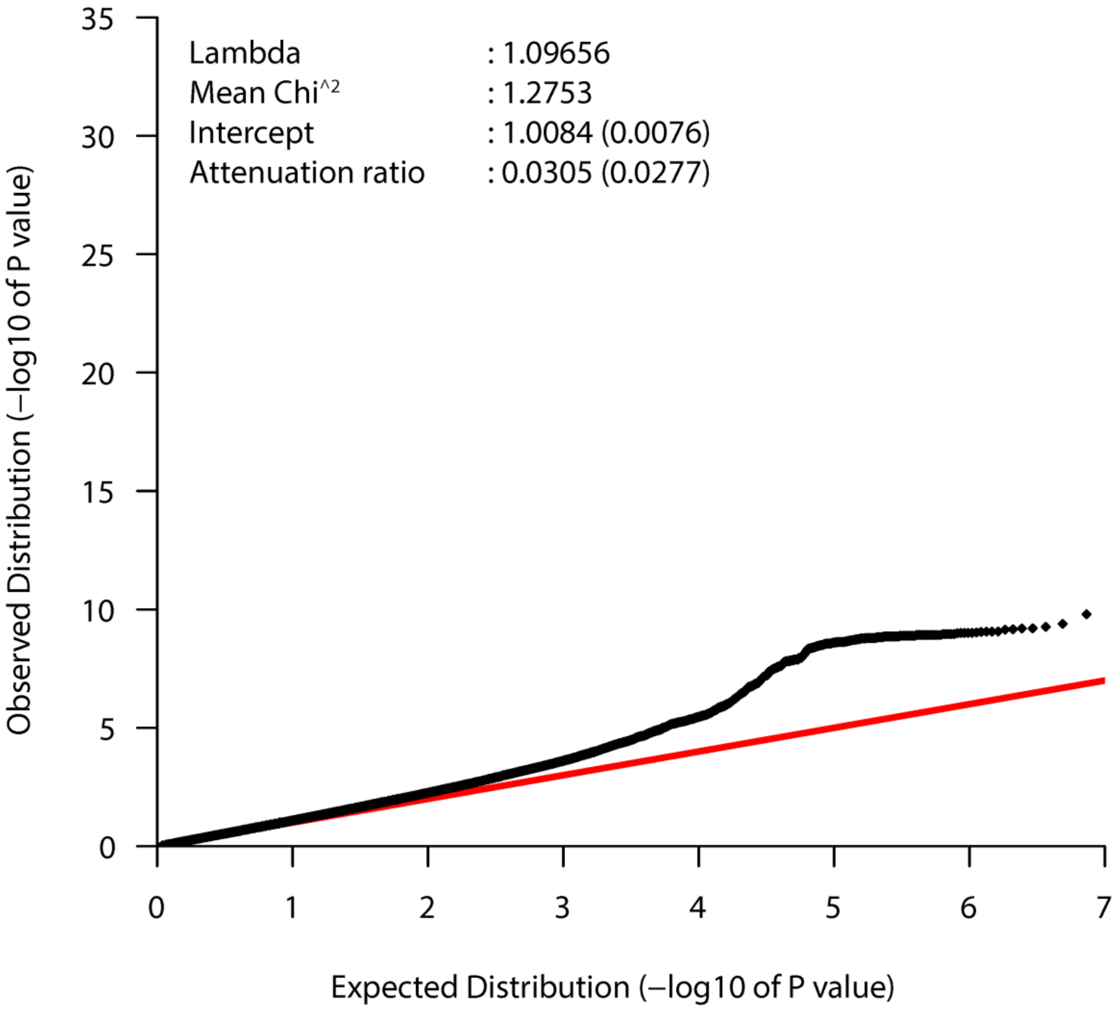

Supplementary Table 1: Correlations among sedentary behaviour traits.

	r television watching	P-value television watching	*r* computer use	P-value computer use	*r* driving	P-value driving
**Television watching**	1	0	-0.0518	1.01E-230	-0.0254	4.81E-57
**Computer use**	-0.0518	1.01E-230	1	0	0.052	1.91E-232
**Driving**	-0.0254	4.81E-57	0.052	1.91E-232	1	0

The associations between sedentary phenotypes were assessed by performing Spearman's rank correlation. A two-sided $P<0.05$ was considered statistically significant. r = correlation coefficient.

Supplementary Table 2: Association of sedentary behaviour traits with possible confounding factors in the observational analyses

outcome	beta	se	OR	OR min (95% CI)	OR plus (95% CI)	P-value	N	$N_{control}$	$N_{outcome}$	(Pseudo) R^2	Z	Estimated Pvalue
Television watching												
Age	1.065	0.008	NA	NA	NA	0	417555	NA	NA	0.0423	135.779	2.78E-4006
Sex	0.013	0.002	1.013	1.009	1.017	4.85E-10	417555	224627	192928	0.0001	6.224	4.85E-10
BMI	0.667	0.005	NA	NA	NA	0	415670	NA	NA	0.0469	142.998	2.68E-4443
Diabetes mellitus	0.301	0.005	1.351	1.338	1.364	0	417555	403078	14477	0.0275	59.777	1.55E-778
Hypertension	0.257	0.003	1.294	1.285	1.302	0	417555	382950	34605	0.0231	74.810	5.67E-1218
Smoking	0.046	0.001	NA	NA	NA	0	417454	NA	NA	0.0111	68.580	5.85E-1024
Physical activity	0.063	0.001	NA	NA	NA	0	416853	NA	NA	0.0234	99.932	2.35E-2171
Alcohol use	0.247	0.019	NA	NA	NA	3.15E-40	349165	NA	NA	0.0005	13.279	3.08E-40
TDI	0.056	0.001	NA	NA	NA	0	417054	NA	NA	0.0079	57.485	3.75E-720
Education years	-1.032	0.005	NA	NA	NA	0	416172	NA	NA	0.1094	226.102	3.60E-11104
HDL	-0.028	0.000	NA	NA	NA	0	356374	NA	NA	0.0127	67.627	9.22E-996
LDL	0.009	0.001	NA	NA	NA	6.56E-24	388595	NA	NA	0.0003	10.084	6.52E-24
TC	0.000	0.001	NA	NA	NA	6.77E-01	389316	NA	NA	0.0000	0.417	6.77E-1
Triglyceride	0.083	0.001	NA	NA	NA	0	389008	NA	NA	0.0158	78.943	5.47E-1356
SBP	1.446	0.018	NA	NA	NA	0	416777	NA	NA	0.0155	80.911	2.62E-1424
DBP	0.453	0.009	NA	NA	NA	0	416779	NA	NA	0.0067	52.964	1.07E-611
Computer use												
Age	-0.457	0.010	NA	NA	NA	0	414927	NA	NA	0.0050	45.867	2.56E-459
Sex	0.231	0.003	1.260	1.253	1.266	0	414927	221999	192928	0.0140	86.113	5.16E-1613
BMI	0.270	0.006	NA	NA	NA	0	413052	NA	NA	0.0049	45.286	8.08E-448
Diabetes mellitus	0.080	0.006	1.083	1.070	1.097	0	414927	400450	14477	0.0013	12.974	1.71E-38
Hypertension	-0.012	0.005	0.988	0.979	0.997	1.11E-02	414927	380322	34605	0.0000	2.540	1.11E-2
Smoking	0.009	0.001	NA	NA	NA	2.67E-29	414825	NA	NA	0.0003	11.238	2.65E-29
Physical activity	0.000	0.001	NA	NA	NA	6.65E-01	414218	NA	NA	0.0000	0.433	6.65E-1

Alcohol use	0.468	0.023	NA	NA	NA	8.51E-91	346888	NA	NA	0.0012	20.213	7.54E-91
TDI	-0.006	0.001	NA	NA	NA	5.84E-07	414425	NA	NA	0.0001	4.997	5.84E-7
Education years	0.670	0.006	NA	NA	NA	0	414927	NA	NA	0.0297	112.733	1.54E-2762
HDL	-0.028	0.001	NA	NA	NA	0	357195	NA	NA	0.0084	55.054	1.02E-660
LDL	-0.010	0.001	NA	NA	NA	1.58E-18	389499	NA	NA	0.0002	8.785	1.57E-18
TC	-0.031	0.001	NA	NA	NA	7.71E-100	390224	NA	NA	0.0012	21.216	6.76E-100
Triglyceride	0.041	0.001	NA	NA	NA	1.07E-207	389918	NA	NA	0.0024	30.773	6.02E-208
SBP	-0.193	0.022	NA	NA	NA	9.34E-18	414164	NA	NA	0.0002	8.582	9.31E-18
DBP	0.201	0.011	NA	NA	NA	6.63E-79	414166	NA	NA	0.0009	18.811	6.14E-79
Driving												
Age	-1.025	0.012	NA	NA	NA	0	414546	NA	NA	0.0179	86.811	3.24E-1639
Sex	0.458	0.004	1.580	1.569	1.592	0	414546	221618	192928	0.0326	123.123	1.02E-3294
BMI	0.366	0.007	NA	NA	NA	0	412656	NA	NA	0.0064	51.586	2.20E-580
Diabetes mellitus	0.022	0.008	1.022	1.007	1.039	5.38E-03	414546	400069	14477	0.0001	2.783	5.38E-3
Hypertension	-0.094	0.006	0.910	0.899	0.921	0	414546	379941	34605	0.0011	15.487	4.25E-54
Smoking	0.012	0.001	NA	NA	NA	4.03E-32	414444	NA	NA	0.0003	11.798	3.99E-32
Physical activity	-0.015	0.001	NA	NA	NA	7.10E-57	413866	NA	NA	0.0006	15.895	6.83E-57
Alcohol use	1.356	0.028	NA	NA	NA	0	346546	NA	NA	0.0069	49.023	2.26E-524
TDI	-0.068	0.001	NA	NA	NA	0	414054	NA	NA	0.0054	47.337	4.49E-489
Education years	-0.039	0.007	NA	NA	NA	4.81E-08	413193	NA	NA	0.0001	5.458	4.80E-8
HDL	-0.045	0.001	NA	NA	NA	0	353924	NA	NA	0.0150	73.531	9.44E-1177
LDL	-0.004	0.001	NA	NA	NA	1.31E-03	385944	NA	NA	0.0000	3.214	1.31E-3
TC	-0.036	0.002	NA	NA	NA	3.97E-93	386656	NA	NA	0.0011	20.476	3.54E-93
Triglyceride	0.060	0.002	NA	NA	NA	0	386356	NA	NA	0.0038	38.139	2.85E-318
SBP	0.389	0.027	NA	NA	NA	9.14E-48	413767	NA	NA	0.0005	14.521	8.90E-48
DBP	0.581	0.013	NA	NA	NA	0	413769	NA	NA	0.0050	45.633	1.17E-454

Associations with potential confounders were assessed using linear regressions analyses. Logistic regression analyses were used in case of binary outcomes. In case logistic regressions were performed, odds ratio's (OR) and 95% confidence intervals (CI). In addition, in case of logistic regressions, we show the pseudo R^2 instead of the actual R^2 (as shown for the linear regression analyses). P-values were on some occasions estimated to be zero using convential methods. We therefore show the Z-values as well as estimated P-values using a mantissa plus exponent method. A two-sided $P<0.05$ was considered statistically significant.

Associations were tested for all a priori chosen confounder used in model 3 of the Cox regression analyses. In addition, associations were tested for traits that were included in the multivariable MR to control for potentially pleiotropic effects in the MR. BMI stands for body mass index, TDI for Townsend Deprivation Index, SBP for systolic blood pressure, DBP for diastolic blood pressure, hypertension for a medical history of hypertension, HDL for high density lipoprotein, LDL for low densitiy lipoprotein and TC for total cholesterol. NA stands for not applicable.

Supplementary Table 3: Association of sedentary behaviour traits with new onset coronary artery disease incidence in the observational analyses.

exposure	Model	beta	se	HR	HR min (95% CI)	HR plus (95% CI)	P-value	Nsubjects	Nfailures
Television watching	Model 1	0.179	0.005	1.196	1.183	1.209	2.71E-227	400364	12136
Computer use	Model 1	-0.006	0.007	0.994	0.980	1.009	0.440	397641	12085
Driving	Model 1	0.029	0.008	1.029	1.013	1.046	0.001	397315	12081
Television watching	Model 2	0.117	0.006	1.124	1.111	1.136	2.37E-88	400364	12136
Computer use	Model 2	-0.026	0.008	0.974	0.959	0.989	0.001	397641	12085
Driving	Model 2	0.024	0.009	1.024	1.007	1.041	0.006	397315	12081
Television watching	Model 3	0.033	0.007	1.034	1.020	1.048	1.15E-06	331402	9755
Computer use	Model 3	-0.008	0.008	0.992	0.976	1.009	0.345	330117	9748
Driving	Model 3	0.011	0.009	1.011	0.993	1.029	0.244	328802	9697

Cox regression analysis was performed to investigate the association between different sedentary behaviours and new onset CAD events using three models. A two-sided P<0.05 was considered statistically significant, no adjustments were made for multiple testing.The models included:
Model 1: univariable analysis
Model 2: age and sex
Model 3: age, sex, body mass index, smoking status, hypertension, diabetes, Townsend deprivation index as proxy for income, physical activity levels, alcohol use per weak and years of education.
HR = Hazard ratio. CI = Confidence interval. Nsubjects = the amount of subjects included in the analyses, Nfailures = the amount of participants who had CAD events.

Supplementary Table 4: Results of the two-sample Mendelian randomization analyses of sedentary traits on coronary artery disease, including the multivariable MR with education.

exposure	method	nsnp	beta	se	P-value	OR	95% CI min	95% CI plus	P-value cut-off
Television	IVW (fixed effects)	127	0.364	0.059	7.28E-10	1.439	1.282	1.616	1.00E-08
Television	IVW (random effects)	127	0.364	0.073	5.63E-07	1.439	1.248	1.660	1.00E-08
Television	MR Egger	127	0.771	0.334	2.26E-02	2.161	1.124	4.156	1.00E-08
Television	IVW (excluding pleiotropic education SNPs)	113	0.363	0.065	1.93E-08	1.437	1.266	1.631	1.00E-08
Television	IVW (excluding pleiotropic SNPs)	93	0.408	0.072	1.26E-08	1.504	1.307	1.731	1.00E-08
Television	MR-PRESSO	127	0.364	0.073	1.85E-06	1.439	1.248	1.660	1.00E-08
Television	MR-PRESSO (Outlier-corrected)	126	0.383	0.070	2.56E-07	1.467	1.278	1.683	1.00E-08
Television	Multivariable (adjusted for education)	126	0.349	0.133	8.70E-03	1.418	1.092	1.840	1.00E-08
Television	Weighted median	127	0.364	0.092	7.82E-05	1.439	1.201	1.723	1.00E-08
Television	Weighted mode	127	0.387	0.251	1.26E-01	1.473	0.900	2.410	1.00E-08
Computer	IVW (fixed effects)	32	-0.212	0.118	7.16E-02	0.809	0.642	1.019	1.00E-08
Computer	IVW (random effects)	32	-0.212	0.153	1.66E-01	0.809	0.599	1.092	1.00E-08
Computer	MR Egger	32	-1.588	1.249	2.13E-01	0.204	0.018	2.364	1.00E-08
Computer	IVW (excluding pleiotropic education SNPs)	28	-0.115	0.128	3.67E-01	0.891	0.693	1.145	1.00E-08
Computer	IVW (excluding pleiotropic SNPs)	27	-0.116	0.131	3.75E-01	0.891	0.689	1.151	1.00E-08
Computer	MR-PRESSO	32	-0.212	0.153	1.76E-01	0.809	0.599	1.092	1.00E-08
Computer	MR-PRESSO (Outlier-corrected)	31	-0.135	0.136	3.30E-01	0.874	0.669	1.141	1.00E-08
Computer	Multivariable (adjusted for education)	32	0.157	0.252	5.34E-01	1.170	0.714	1.916	1.00E-08
Computer	Weighted median	32	-0.204	0.172	2.35E-01	0.816	0.582	1.142	1.00E-08
Computer	Weighted mode	32	-0.222	0.316	4.88E-01	0.801	0.431	1.489	1.00E-08
Driving	IVW (fixed effects)	4	0.975	0.343	4.46E-03	2.651	1.354	5.191	1.00E-08
Driving	IVW (random effects)	4	0.975	0.343	4.46E-03	2.651	1.354	5.191	1.00E-08
Driving	MR Egger	4	-0.721	2.849	8.24E-01	0.486	0.002	129.317	1.00E-08
Driving	IVW (excluding pleiotropic education SNPs)	3	1.053	0.402	8.86E-03	2.866	1.303	6.303	1.00E-08

Driving	IVW (excluding pleiotropic SNPs)	2	0.688	0.480	1.52E-01	1.990	0.776	5.103	1.00E-08
Driving	MR-PRESSO	4	0.975	0.285	4.17E-02	2.651	1.517	4.633	1.00E-08
Driving	MR-PRESSO (Outlier-corrected)	NA	NA	NA	NA	NA	NA	NA	1.00E-08
Driving	Multivariable (adjusted for education)	4	1.196	0.474	1.17E-02	3.306	1.305	8.378	1.00E-08
Driving	Weighted median	4	0.743	0.407	6.78E-02	2.102	0.947	4.667	1.00E-08
Driving	Weighted mode	4	0.727	0.542	2.72E-01	2.070	0.716	5.983	1.00E-08
Television	IVW (fixed effects)	182	0.359	0.052	3.98E-12	1.431	1.293	1.584	5.00E-08
Television	IVW (random effects)	182	0.359	0.063	9.87E-09	1.431	1.266	1.618	5.00E-08
Television	MR Egger	182	0.623	0.276	2.50E-02	1.865	1.086	3.202	5.00E-08
Television	IVW (excluding pleiotropic education SNPs)	168	0.357	0.055	1.03E-10	1.429	1.282	1.592	5.00E-08
Television	IVW (excluding pleiotropic SNPs)	148	0.387	0.059	7.64E-11	1.473	1.311	1.655	5.00E-08
Television	MR-PRESSO	182	0.359	0.063	4.07E-08	1.431	1.266	1.618	5.00E-08
Television	MR-PRESSO (Outlier-corrected)	181	0.373	0.061	5.93E-09	1.452	1.288	1.637	5.00E-08
Television	Multivariable (adjusted for education)	179	0.315	0.107	3.19E-03	1.370	1.111	1.689	5.00E-08
Television	Weighted median	182	0.364	0.080	5.74E-06	1.439	1.230	1.685	5.00E-08
Television	Weighted mode	182	0.422	0.264	1.12E-01	1.525	0.909	2.560	5.00E-08
Computer	IVW (fixed effects)	47	-0.240	0.101	1.79E-02	0.787	0.645	0.960	5.00E-08
Computer	IVW (random effects)	47	-0.240	0.131	6.70E-02	0.787	0.609	1.017	5.00E-08
Computer	MR Egger	47	-1.130	0.874	2.03E-01	0.323	0.058	1.793	5.00E-08
Computer	IVW (excluding pleiotropic education SNPs)	43	-0.175	0.108	1.04E-01	0.839	0.680	1.037	5.00E-08
Computer	IVW (excluding pleiotropic SNPs)	41	-0.175	0.110	1.13E-01	0.840	0.676	1.042	5.00E-08
Computer	MR-PRESSO	47	-0.247	0.129	6.10E-02	0.781	0.607	1.005	5.00E-08
Computer	MR-PRESSO (Outlier-corrected)	45	-0.149	0.110	1.82E-01	0.862	0.694	1.069	5.00E-08
Computer	Multivariable (adjusted for education)	47	-0.057	0.210	7.87E-01	0.945	0.625	1.427	5.00E-08
Computer	Weighted median	47	-0.224	0.154	1.47E-01	0.799	0.591	1.082	5.00E-08
Computer	Weighted mode	47	-0.242	0.316	4.47E-01	0.785	0.423	1.458	5.00E-08
Driving	IVW (fixed effects)	5	0.780	0.314	1.31E-02	2.182	1.178	4.039	5.00E-08
Driving	IVW (random effects)	5	0.780	0.318	1.41E-02	2.182	1.170	4.068	5.00E-08
Driving	MR Egger	5	-0.540	3.235	8.78E-01	0.583	0.001	330.296	5.00E-08
Driving	IVW (excluding pleiotropic education SNPs)	4	0.784	0.358	2.87E-02	2.189	1.085	4.417	5.00E-08

Driving	IVW (excluding pleiotropic SNPs)	3	0.435	0.410	2.89E-01	1.544	0.691	3.449	5.00E-08
Driving	MR-PRESSO	5	0.780	0.318	7.02E-02	2.182	1.170	4.068	5.00E-08
Driving	MR-PRESSO (Outlier-corrected)	NA	NA	NA	NA	NA	NA	NA	5.00E-08
Driving	Multivariable (adjusted for education)	5	0.888	0.540	9.97E-02	2.431	0.844	7.002	5.00E-08
Driving	Weighted median	5	0.728	0.422	8.45E-02	2.071	0.906	4.735	5.00E-08
Driving	Weighted mode	5	0.721	0.522	2.39E-01	2.057	0.740	5.719	5.00E-08
Television	IVW (fixed effects)	214	0.354	0.049	3.30E-13	1.425	1.296	1.568	1.00E-07
Television	IVW (random effects)	214	0.354	0.058	7.88E-10	1.425	1.273	1.596	1.00E-07
Television	MR Egger	214	0.564	0.256	2.85E-02	1.758	1.065	2.904	1.00E-07
Television	IVW (excluding pleiotropic education SNPs)	200	0.352	0.052	8.48E-12	1.422	1.286	1.574	1.00E-07
Television	IVW (excluding pleiotropic SNPs)	180	0.378	0.055	6.69E-12	1.459	1.310	1.625	1.00E-07
Television	MR-PRESSO	214	0.354	0.058	3.83E-09	1.425	1.273	1.596	1.00E-07
Television	MR-PRESSO (Outlier-corrected)	213	0.367	0.056	5.37E-10	1.444	1.293	1.613	1.00E-07
Television	Multivariable (adjusted for education)	211	0.342	0.099	5.23E-04	1.408	1.161	1.709	1.00E-07
Television	Weighted median	214	0.364	0.074	9.31E-07	1.439	1.244	1.665	1.00E-07
Television	Weighted mode	214	0.433	0.246	7.92E-02	1.542	0.953	2.497	1.00E-07
Computer	IVW (fixed effects)	56	-0.294	0.095	1.99E-03	0.745	0.618	0.898	1.00E-07
Computer	IVW (random effects)	56	-0.294	0.124	1.76E-02	0.745	0.585	0.950	1.00E-07
Computer	MR Egger	56	-1.012	0.800	2.11E-01	0.364	0.076	1.744	1.00E-07
Computer	IVW (excluding pleiotropic education SNPs)	52	-0.244	0.100	1.50E-02	0.784	0.644	0.954	1.00E-07
Computer	IVW (excluding pleiotropic SNPs)	50	-0.247	0.103	1.60E-02	0.781	0.639	0.955	1.00E-07
Computer	MR-PRESSO	56	-0.300	0.122	1.72E-02	0.741	0.584	0.941	1.00E-07
Computer	MR-PRESSO (Outlier-corrected)	53	-0.179	0.102	8.40E-02	0.836	0.684	1.020	1.00E-07
Computer	Multivariable (adjusted for education)	56	-0.168	0.197	3.95E-01	0.846	0.575	1.244	1.00E-07
Computer	Weighted median	56	-0.237	0.144	1.01E-01	0.789	0.595	1.047	1.00E-07
Computer	Weighted mode	56	-0.262	0.307	3.96E-01	0.769	0.422	1.404	1.00E-07
Driving	IVW (fixed effects)	7	0.831	0.262	1.49E-03	2.296	1.375	3.835	1.00E-07
Driving	IVW (random effects)	7	0.831	0.262	1.49E-03	2.296	1.375	3.835	1.00E-07
Driving	MR Egger	7	0.424	1.392	7.73E-01	1.528	0.100	23.407	1.00E-07
Driving	IVW (excluding pleiotropic education SNPs)	6	0.843	0.285	3.13E-03	2.324	1.328	4.066	1.00E-07

Driving	IVW (excluding pleiotropic SNPs)	5	0.655	0.310	3.45E-02	1.925	1.049	3.531	1.00E-07
Driving	MR-PRESSO	7	0.831	0.219	9.09E-03	2.296	1.494	3.531	1.00E-07
Driving	MR-PRESSO (Outlier-corrected)	NA	NA	NA	NA	NA	NA	NA	1.00E-07
Driving	Multivariable (adjusted for education)	7	0.938	0.326	4.05E-03	2.556	1.348	4.846	1.00E-07
Driving	Weighted median	7	0.767	0.333	2.12E-02	2.153	1.121	4.135	1.00E-07
Driving	Weighted mode	7	0.757	0.445	1.39E-01	2.132	0.892	5.097	1.00E-07
Television	IVW (fixed effects)	372	0.312	0.040	6.20E-15	1.367	1.263	1.478	1.00E-06
Television	IVW (random effects)	372	0.312	0.047	3.23E-11	1.367	1.246	1.499	1.00E-06
Television	MR Egger	372	0.460	0.197	2.03E-02	1.584	1.076	2.332	1.00E-06
Television	IVW (excluding pleiotropic education SNPs)	358	0.308	0.042	1.46E-13	1.360	1.254	1.476	1.00E-06
Television	IVW (excluding pleiotropic SNPs)	338	0.319	0.043	1.72E-13	1.376	1.264	1.498	1.00E-06
Television	MR-PRESSO	372	0.312	0.047	1.15E-10	1.367	1.246	1.499	1.00E-06
Television	MR-PRESSO (Outlier-corrected)	371	0.323	0.045	3.66E-12	1.381	1.265	1.509	1.00E-06
Television	Multivariable (adjusted for education)	367	0.259	0.078	9.39E-04	1.295	1.111	1.510	1.00E-06
Television	Weighted median	372	0.320	0.061	1.91E-07	1.377	1.221	1.553	1.00E-06
Television	Weighted mode	372	0.381	0.210	7.05E-02	1.464	0.970	2.210	1.00E-06
Computer	IVW (fixed effects)	112	-0.216	0.073	2.97E-03	0.805	0.698	0.929	1.00E-06
Computer	IVW (random effects)	112	-0.216	0.084	1.02E-02	0.805	0.683	0.950	1.00E-06
Computer	MR Egger	112	-0.358	0.462	4.40E-01	0.699	0.282	1.730	1.00E-06
Computer	IVW (excluding pleiotropic education SNPs)	108	-0.183	0.075	1.46E-02	0.833	0.719	0.964	1.00E-06
Computer	IVW (excluding pleiotropic SNPs)	106	-0.183	0.076	1.57E-02	0.832	0.717	0.966	1.00E-06
Computer	MR-PRESSO	112	-0.212	0.083	1.23E-02	0.809	0.687	0.952	1.00E-06
Computer	MR-PRESSO (Outlier-corrected)	110	-0.162	0.077	3.91E-02	0.851	0.731	0.990	1.00E-06
Computer	Multivariable (adjusted for education)	112	-0.132	0.115	2.50E-01	0.876	0.700	1.098	1.00E-06
Computer	Weighted median	112	-0.206	0.104	4.86E-02	0.814	0.663	0.999	1.00E-06
Computer	Weighted mode	112	-0.214	0.269	4.28E-01	0.807	0.476	1.369	1.00E-06
Driving	IVW (fixed effects)	26	0.238	0.157	1.29E-01	1.269	0.933	1.727	1.00E-06
Driving	IVW (random effects)	26	0.238	0.227	2.93E-01	1.269	0.814	1.979	1.00E-06
Driving	MR Egger	26	0.675	1.154	5.64E-01	1.964	0.204	18.861	1.00E-06
Driving	IVW (excluding pleiotropic education SNPs)	25	0.206	0.162	2.02E-01	1.229	0.895	1.687	1.00E-06

Driving	IVW (excluding pleiotropic SNPs)	23	0.048	0.169	7.78E-01	1.049	0.753	1.461	1.00E-06
Driving	MR-PRESSO	26	0.135	0.223	5.49E-01	1.145	0.739	1.773	1.00E-06
Driving	MR-PRESSO (Outlier-corrected)	NA	NA	NA	NA	NA	NA	NA	1.00E-06
Driving	Multivariable (adjusted for education)	26	-0.060	0.275	8.29E-01	0.942	0.549	1.616	1.00E-06
Driving	Weighted median	26	0.324	0.257	2.09E-01	1.382	0.835	2.290	1.00E-06
Driving	Weighted mode	26	0.557	0.469	2.46E-01	1.745	0.696	4.377	1.00E-06
Television	IVW (fixed effects)	717	0.291	0.032	2.94E-20	1.338	1.257	1.423	1.00E-05
Television	IVW (random effects)	717	0.291	0.037	3.28E-15	1.338	1.244	1.438	1.00E-05
Television	MR Egger	717	0.207	0.133	1.21E-01	1.230	0.947	1.596	1.00E-05
Television	IVW (excluding pleiotropic education SNPs)	703	0.287	0.032	6.34E-19	1.332	1.251	1.419	1.00E-05
Television	IVW (excluding pleiotropic SNPs)	683	0.293	0.033	8.64E-19	1.340	1.256	1.430	1.00E-05
Television	MR-PRESSO	717	0.291	0.037	1.22E-14	1.338	1.244	1.438	1.00E-05
Television	MR-PRESSO (Outlier-corrected)	713	0.292	0.035	3.32E-16	1.339	1.251	1.434	1.00E-05
Television	Multivariable (adjusted for education)	706	0.175	0.058	2.73E-03	1.191	1.062	1.336	1.00E-05
Television	Weighted median	717	0.281	0.049	8.12E-09	1.324	1.203	1.456	1.00E-05
Television	Weighted mode	717	0.277	0.165	9.34E-02	1.320	0.955	1.824	1.00E-05
Computer	IVW (fixed effects)	272	-0.105	0.052	4.51E-02	0.901	0.813	0.998	1.00E-05
Computer	IVW (random effects)	272	-0.105	0.061	8.75E-02	0.901	0.799	1.016	1.00E-05
Computer	MR Egger	268	-0.085	0.053	1.10E-01	0.919	0.828	1.019	1.00E-05
Computer	IVW (excluding pleiotropic education SNPs)	266	-0.084	0.053	1.17E-01	0.920	0.828	1.021	1.00E-05
Computer	IVW (excluding pleiotropic SNPs)	272	-0.159	0.253	5.31E-01	0.853	0.520	1.401	1.00E-05
Computer	MR-PRESSO	272	-0.102	0.060	9.25E-02	0.903	0.803	1.017	1.00E-05
Computer	MR-PRESSO (Outlier-corrected)	270	-0.073	0.057	2.01E-01	0.929	0.831	1.040	1.00E-05
Computer	Multivariable (adjusted for education)	268	0.013	0.076	8.66E-01	1.013	0.873	1.176	1.00E-05
Computer	Weighted median	272	-0.153	0.079	5.37E-02	0.858	0.734	1.002	1.00E-05
Computer	Weighted mode	272	-0.227	0.254	3.72E-01	0.797	0.485	1.310	1.00E-05
Driving	IVW (fixed effects)	96	0.151	0.091	9.65E-02	1.163	0.973	1.391	1.00E-05
Driving	IVW (random effects)	96	0.151	0.097	1.20E-01	1.163	0.961	1.408	1.00E-05
Driving	MR Egger	96	-0.250	0.328	4.48E-01	0.779	0.409	1.482	1.00E-05
Driving	IVW (excluding pleiotropic education SNPs)	95	0.139	0.092	1.30E-01	1.149	0.960	1.376	1.00E-05

Driving	IVW (excluding pleiotropic SNPs)	93	0.089	0.093	3.39E-01	1.093	0.911	1.312	1.00E-05
Driving	MR-PRESSO	96	0.117	0.098	2.34E-01	1.124	0.928	1.362	1.00E-05
Driving	MR-PRESSO (Outlier-corrected)	NA	NA	NA	NA	NA	NA	NA	1.00E-05
Driving	Multivariable (adjusted for education)	95	0.022	0.104	8.31E-01	1.022	0.834	1.254	1.00E-05
Driving	Weighted median	96	0.158	0.139	2.54E-01	1.171	0.893	1.537	1.00E-05
Driving	Weighted mode	96	-0.154	0.378	6.84E-01	0.857	0.408	1.797	1.00E-05

Variants at $P < 1 \times 10^{-8}$ were used for the main analysis. IVW stands for inverse variance weighted. A two-sided P<0.05 was considered statistically significant, no adjustments were made for multiple testing.
N=417,555, N=414,546, N=414,546 biologically independent individuals from the UK Biobank were used to obtain effect estimates of the genetic variants associated with television watching, computer use and driving, respectively. N=184,305 (60,801 cases, 123,504 controls) individuals were used to obtain effect estimates of the genetic variants associated with CAD.
Nsps = number of snps. CI = confidence interval.

Supplementary Table 5: Heterogeneity (I^2, Cochran's Q, Rucker's Q and Q-Q'), pleiotropy (MR-Egger intercept) and weak instrument statistics in the MR-Egger analyses (I^2_{GX}) in the examined associations between sedentary behaviours and coronary artery disease.

exposure	I^2			Cochran's Q			Rucker's Q			Q-Q'			MR Egger intercept			I^2_{GX}	Cut-off
	index	95% CI min	95% CI max	Q	df	P-value	Q	df	P-value	Q-Q'	df	P-value	Inter-cept	se	P-value		
Television	0.340	0.178	0.471	190.988	126	1.67E-04	188.635	125	2.05E-04	2.353	1	0.125	-0.007	0.005	0.214	0.978	1E-08
Computer	0.410	0.098	0.614	52.513	31	9.30E-03	50.442	30	1.12E-02	2.071	1	0.150	0.022	0.020	0.276	0.975	1E-08
Driving	0.000	0.000	0.000	2.070	3	5.60E-01	1.710	2	4.30E-01	0.360	1	0.550	0.026	0.043	0.610	0.000	1E-08
Television	0.317	0.178	0.433	265.089	181	4.61E-05	263.666	180	4.79E-05	1.423	1	0.233	-0.004	0.004	0.326	0.976	5E-08
Computer	0.401	0.149	0.579	76.815	46	2.90E-03	75.047	45	3.30E-03	1.768	1	0.184	0.014	0.013	0.309	0.974	5E-08
Driving	0.023	0.000	0.797	4.090	4	3.90E-01	3.880	3	2.80E-01	0.220	1	0.640	0.020	0.049	0.709	0.964	5E-08
Television	0.287	0.152	0.401	298.850	213	9.38E-05	297.853	212	9.12E-05	0.997	1	0.318	-0.003	0.004	0.400	0.974	1E-07
Computer	0.410	0.187	0.573	93.287	55	9.70E-04	91.883	54	1.00E-03	1.403	1	0.236	0.011	0.012	0.368	0.974	1E-07
Driving	0.000	0.000	0.000	4.220	6	6.47E-01	4.131	5	5.31E-01	0.089	1	0.766	0.006	0.022	0.778	0.966	1E-07
Television	0.276	0.174	0.366	512.558	371	1.46E-06	511.736	370	1.39E-06	0.823	1	0.364	-0.002	0.003	0.441	0.970	1E-06
Computer	0.252	0.048	0.412	148.440	111	1.02E-02	148.309	110	8.80E-03	0.131	1	0.717	0.002	0.007	0.756	0.968	1E-06
Driving	0.520	0.249	0.694	52.109	25	1.16E-03	51.788	24	8.30E-04	0.322	1	0.571	-0.006	0.015	0.703	0.965	1E-06
Television	0.270	0.197	0.336	980.508	716	1.54E-10	979.915	715	1.42E-10	0.592	1	0.442	0.001	0.002	0.511	0.964	1E-05
Computer	0.273	0.152	0.377	370.000	271	3.90E-05	370.000	270	3.40E-05	0.067	1	0.800	0.001	0.003	0.826	0.961	1E-05
Driving	0.127	0.000	0.330	108.780	95	1.60E-01	106.920	94	1.70E-01	1.860	1	0.170	0.006	0.005	0.204	0.957	1E-05

An I^2 index >25% and Cochran's Q an one-sided P-value of <0.05 were considered as an indication of heterogeneity and, as a consequence, of pleiotropy in the from the inverse variance weighted fixed effects model. A significant difference (one-sided P<0.05) between the Cochran's Q and Rucker's Q (Q-Q') was considered to indicate the MR-Egger test to be a better method to study the genetic association between the particular exposure and outcome. An MR-Egger's intercept of zero, tested using a single-sided P-value threshold of >0.05, was considered to provide evidence for absence of pleiotropic bias. An I^2_{GX} of >95% was considered low risk of measurement error within the MR-Egger test.
CI = confidence interval. Df = Degrees of freedom. Cutoff = the P-value cut-off value for the SNPs to be included in the Mendelian randomization analyses.

Supplementary Table 6: SNPs excluded from the outlier corrected MR-PRESSO analyses between sedentary behaviours and coronary artery disease.

OutlierSNP	RSSobs	P-value	P-value cut-off
Television watching on CAD			
rs7248205	1.55E-03	<0.0473	1.00E-08
rs7248205	1.54E-03	<0.0469	5,00E-08
rs7248205	1.54E-03	<0.0471	1.00E-07
rs4420638	9.30E-03	<0.0473	1.00E-06
rs1905339	1.81E-03	<0.0477	1.00E-05
rs4420638	9.23E-03	<0.0477	1.00E-05
rs550057	3.54E-03	<0.0477	1.00E-05
rs7248205	1.46E-03	0.0477	1.00E-05
Computer use on CAD			
rs11708955	0.001578495	<0.05	1.00E-08
rs11708955	0.001507788	<0.05	5.00E-08
rs4702	0.001229142	<0.05	5.00E-08
rs11708955	0.001435054	0.05	1.00E-07
rs4702	0.001180032	<0.05	1.00E-07
rs55836224	0.001514467	0.05	1.00E-07
rs11708955	0.001518	<0.05	1.00E-06
rs4702	0.001242084	<0.05	1.00E-06
rs11708955	0.001578495	<0.05	1.00E-05
rs11708955	0.001639525	0.1	1.00E-05
rs6857	0.0059279	<0.05	1.00E-05
Driving on CAD			
NA	NA	NA	1.00E-08
NA	NA	NA	5,00E-08
NA	NA	NA	1.00E-07
NA	NA	NA	1.00E-06
NA	NA	NA	1.00E-05

Excluded variants are shown for the analyses between television watching, computer use, driving and CAD for every P-value cutoff. MR-PRESSO does not return exact P-values in case variants are filtered and therefore non-exact P-values are shown.
OutlierSNP= Single nucleotide polymorphism removed from the analysis by MR-PRESSO. RSSobs= observed residual sum of squares. P-value cutoff = the cut-off value for the SNPs to be included in the Mendelian randomization analyses.

Supplementary Table 7: Potentially pleiotropic SNPs in the Mendelian randomization between sedentary behaviours coronary artery disease.

SNP	P-value	Pleiotropic effect(s)	Reference(s)
**SNPs removed for the analysis between television watching and CAD**			
rs3796386	3.20E-33	Educational attainment	Davies G, 2016; Okbay A, 2016
	3.20E-33	Menarche (age at onset)	Perry JR, 2014; Elks CE, 2010
rs12554512	3.80E-21	Educational attainment	Davies G, 2016;
	3.80E-21	Bipolar disorder	Hou L, 2016
rs10189857	6.20E-21	Schizophrenia	Goes FS, 2015
rs9718104	9.30E-19	Bipolar disorder	Kerner B, 2011
rs6905544	8.50E-18	Gut microbiota (bacterial taxa)	Bonder MJ, 2016
	8.50E-18	Educational attainment	Okbay A, 2016
rs11689199	5.50E-17	Educational attainment	Okbay A, 2016; Rietveld 2013
rs17379561	1.10E-16	Educational attainment	Okbay A, 2016;
rs1243182	2.00E-15	Ovarian cancer	Pharoah PD, 2013
		Epithelial ovarian cancer	Kuchenbaecker KB, 2015
rs262890	3.20E-15	Educational attainment	Okbay A, 2016;
rs2616830	2.90E-14	Educational attainment	Rietveld, 2013
rs9964724	3.30E-14	Educational attainment	Davies G, 2016; Okbay A, 2016;
		Neuroticism	Okbay A, 2016;
rs801733	7.30E-14	Gout	Nakayama A, 2016;Matsuo H, 2015
rs870151	8.80E-13	Fibrinogen levels	de Vries PS, 2017
rs10772643	1.30E-12	Educational attainment	Okbay A, 2016;
rs13107325	1.50E-12	HDL cholesterol	Surakka I, 2015; Willer CJ, 2013; Teslovich TM, 2010
		Blood pressure	Wain LV, 2011
		Systolic blood pressure	Ehret GB, 2011
		Diastolic blood pressure	Ehret GB, 2011
		Hypertension	Ehret GB, 2011
		Body mass index	Locke AE, 2015; Speliotes EK, 2010
		Hypertension	Ehret GB, 2011
		NT-proBNP levels in acute coronary syndrome	Johansson Å, 2016
		Childhood body mass index	Felix JF, 2015
		Schizophrenia	Goes FS, 2015
rs749671	2.70E-12	Hip circumference	Shungin D, 2015
		Blood	Cooper GM, 2008
		Triglyceride levels	Spracklen CN, 2017
		Body mass index	Locke AE, 2015
		Parkinson's disease	Nalls MA, 2015
		Warfarin maintenance dose	Parra EJ, 2015; Cha PC, 2010; Takeuchi F, 2009
rs3754970	4.80E-12	Educational attainment	Rietveld, 2013
rs11810109	5.40E-12	Educational attainment	Okbay A, 2016;

rs2447098	6.80E-12	Autism spectrum disorder	Kuo PH, 2015
rs6673341	2.20E-11	Obesity-related traits	Comuzzie AG, 2012
rs263771	3.10E-11	Malaria	Band G, 2013
rs7189927	3.40E-11	Obesity	Berndt SI, 2013
		Hip circumference	Shungin D, 2015
		Body mass index	Locke AE, 2015; Willer CJ, 2008; Speliotes, 2010
		Body fat percentage	Lu Y, 2016
		Inflammatory bowel disease	Liu JZ, 2015; Imielinski M, 2009
		Weight	Thorleifsson G, 2008
		Waist circumference	Shungin D, 2015
		Educational attainment	Okbay A, 2016; Rietveld, 2013
rs10786658	4.60E-11	Educational attainment	Okbay A, 2016;
rs11714337	4.70E-11	Educational attainment	Okbay A, 2016;
rs7043521	6.50E-11	Educational attainment	Rietveld, 2013
rs62332760	8.00E-11	Height	He M, 2014; Berndt SI, 2013;
		Inflammatory bowel disease	Liu JZ, 2015
		Ulcerative colitis	Liu JZ, 2015
rs3135044	8.70E-11	Height	Wood AR, 2014
		Psychosis (atypical)	Kanazawa T
rs2584597	2.90E-10	Height	Wood AR, 2014; Carty CL, 2011; N'Diaye A, 2011; Gudbjartsson DF, 2008
		Hip circumference	Shungin D, 2015
		Body mass index	Locke AE, 2015
rs7248205	3.40E-10	Height	He M, 2014
rs1913808	3.90E-10	Educational attainment	Rietveld, 2013
		Rheumatoid arthritis	Jiang L, 2014
rs8756	5.30E-10	Height	Wood AR, 2014; He M, 2014; Berndt SI, 2013; Carty CL, 2011; Lango Allen H, 2010; Liu JZ, 2010; Soranzo N 2009; Gudbjartsson DF, 2008; Weedon MN, 2008; Lettre G, 2008
		Birth length	van der Valk RJ, 2014
		Infant length	van der Valk RJ, 2014
		Pulse pressure	Warren, 2017
		Hip circumference	Shungin D, 2015
		Brain structure	Stein JL, 2012
		Head circumference (infant)	Taal HR, 2012
		Birth weight	Horikoshi M, 2012
rs71658797	6.30E-10	Psoriasis	Tsoi LC, 2017
rs648044	1.00E-09	Non-glioblastoma glioma	Melin BS, 2017; Kinnersley B, 2015
rs10876864	1.00E-09	Vitiligo	Tang XF, 2012
		Inflammatory skin disease	Baurecht H, 2015
		Rheumatoid arthritis	Okada Y, 2013
		Type 1 diabetes	WTCCC, 2007

rs6141814	1.30E-09	Ulcerative colitis	Liu JZ, 2015
		Inflammatory bowel disease	Liu JZ, 2015; Jostins L, 2012
rs111901094	2.00E-09	Height	Wood AR, 2014
rs9834970	3.30E-09	Bipolar disorder	Charney AW, 2017; Ikeda M, 2017; Hou L, 2016; Goes FS, 2012; Chen DT 2011
		Autism spectrum disorder, attention deficit-hyperactivity disorder, bipolar disorder, major depressive disorder, and schizophrenia (combined)	Smoller JW, 2013
		Schizophrenia or bipolar disorder	Ruderfer DM, 2013
		Subjective response to lithium treatment	Song J, 2015
rs2045147	5.90E-09	Extraversion	Lo MT, 2016
rs78394231	9.70E-09	Airway responsiveness in COPD	Hansel NN, 2014
rs80270210	4.00E-06	Waist-to-hip ratio	Shungin D, 2015
		Triglycerides	Spracklen CN, 2017; Willer CJ, 2013; Teslovich TM, 2010
		HDL cholesterol	Teslovich TM, 2010
		Adiponectin levels	Wu Y, 2013
**SNPs removed for the analysis between computer use and CAD**			
rs4977839	4.70E-19	Educational attainment	Okbay A, 2016; Davies G, 2016
		Bipolar disorder	Hou L, 2016
rs13262595	1.20E-12	Schizophrenia	Goes FS, 2015; Ripke S, 2014
rs11708955	4.20E-11	Pediatric autoimmune diseases	Li YR, 2015
		Gut microbiota (functional units)	Bonder MJ, 2016
		Cognitive function	Davies G, 2015
		Educational attainment	Okbay A, 2016
			Davies G, 2015
			Rietveld CA, 2014
		Inflammatory bowel disease	de Lange KM, 2017; Liu JZ, 2015; Jostins L, 2012
		Crohn's disease	Franke A, 2010; Liu JZ, 2015; Barrett JC, 2009; Parkes M, 2007 ;WTCCC, 2007
		Ulcerative colitis	de Lange KM, 2017; Liu JZ, 2015; Julià A, 2014; Anderson CA, 2011; McGovern DP, 2010; Barrett JC, 2009
		Primary sclerosing cholangitis	Ji SG, 2016; Melum E 2010
rs1448355	1.30E-10	Educational attainment	Okbay A, 2016
rs6129084	9.30E-10	Educational attainment	Okbay A, 2016
rs9477970	1.00E-08	Cognitive function	Davies G, 2015
		Age at voice drop	Pickrell JK, 2016

SNPs removed for the analysis between driving and CAD			
rs1198575	2.00E-11	Educational attainment	Rietveld CA, 2014
rs4765541	5.10E-09	Adiponectin levels	Wu Y, 2013
		Triglyceride levels	Spracklen CN, 2017; & Willer CJ, 2013
		HDL cholesterol	Willer CJ, 2013; Teslovich, TM
		WHR adjusted for BMI	Shungin D, 2015
rs10189857	2.40E-07	Schizophrenia	Goes FS, 2015

Genetic variants in LD >0.8 with previously established variants were removed from the fixed effects meta-analysis in case they were associated with a) education traits b) all traits. SNP denotes single nucleotide polymorphism

Supplementary Table 8: Results from TwoSample Mendelian randomization analysis between education and sedentary behaviours traits.

exposure	outcome	method	nsnp	beta	se	P-value	OR	95% CI min	95% CI plus
Education	Television	Inverse variance weighted (fixed effects)	71	-0.535	0.016	3.40E-261	0.585	0.568	0.604
Education	Television	MR Egger	71	-0.657	0.145	2.46E-05	0.518	0.390	0.689
Education	Television	MR-PRESSO	71	-0.535	0.032	2.51E-26	0.585	0.550	0.623
Education	Television	MR-PRESSO (Outlier-corrected)	67	-0.508	0.028	6.60E-27	0.602	0.569	0.637
Education	Television	Weighted median	71	-0.483	0.032	2.29E-51	0.617	0.579	0.657
Education	Television	Weighted mode	71	-0.538	0.093	2.02E-07	0.584	0.486	0.701
Education	Computer	Inverse variance weighted (fixed effects)	71	0.261	0.016	2.11E-61	1.298	1.259	1.339
Education	Computer	MR Egger	71	0.504	0.146	9.25E-04	1.655	1.244	2.201
Education	Computer	MR-PRESSO	71	0.261	0.032	1.33E-11	1.298	1.219	1.383
Education	Computer	MR-PRESSO (Outlier-corrected)	62	0.239	0.024	1.14E-14	1.269	1.212	1.329
Education	Computer	Weighted median	71	0.234	0.028	1.85E-16	1.264	1.195	1.336
Education	Computer	Weighted mode	71	0.247	0.060	9.33E-05	1.280	1.139	1.438
Education	Driving	Inverse variance weighted (fixed effects)	71	-0.077	0.016	1.37E-06	0.926	0.897	0.955
Education	Driving	MR Egger	71	-0.136	0.088	0.126231	0.873	0.735	1.037
Education	Driving	MR-PRESSO	71	-0.077	0.019	0.000137	0.926	0.892	0.961
Education	Driving	MR-PRESSO (Outlier-corrected)	70	-0.072	0.018	0.000201	0.931	0.898	0.965
Education	Driving	Weighted median	71	-0.063	0.024	0.008228	0.939	0.896	0.984
Education	Driving	Weighted mode	71	-0.033	0.052	0.520389	0.967	0.874	1.070

Variants at $P < 5 \times 10^{-8}$ were used for the MR analysis. A two-sided $P < 0.05$ was considered statistically significant for the Mendelian randomization analyses, no correction was made for multiple testing.
Nsps = number of snps. OR = odds ratio. CI = confidence interval.

Supplementary Table 9: Heterogeneity (I^2, Cochran's Q, Rucker's Q and Q-Q'), pleiotropy (MR-Egger intercept) and weak instrument statistics in the MR-Egger analysis (I^2_{GX}) in the examined associations between education and sedentary behaviours.

outcome	I^2			Cochran's Q			Rucker's Q			Q-Q'			MR Egger intercept			I^2_{GX}
	index	95% CI min	95% CI max	Q	df	P-value	Q	df	P-value	Q	df	P-value	Intercept	se	P-value	
Television	0.762	0.702	0.810	290	70	3.00E-29	290	69	4.80E-29	3.1	1	7.80E-02	0.002	0.003	0.394	0.974
Computer	0.761	0.701	0.810	290	70	3.80E-29	280	69	1.80E-27	12	1	5.60E-04	-0.005	0.003	0.092	0.974
Driving	0.302	0.060	0.481	100	70	1.03E-02	100	69	9.40E-03	0.6732	1	4.12E-01	0.001	0.002	0.497	0.974

An I^2 index >25% and Cochran's Q an one-sided P-value of <0.05 were considered as an indication of heterogeneity and, as a consequence, of pleiotropy in the from the inverse variance weighted fixed effects model. A significant difference (one-sided P<0.05) between the Cochran's Q and Rucker's Q (Q-Q') was considered to indicate the MR-Egger test to be a better method to study the genetic association between the particular exposure and outcome. An MR-Egger's intercept of zero, tested using a single-sided P-value threshold of >0.05, was considered to provide evidence for absence of pleiotropic bias. An I^2_{GX} of >95% was considered low risk of measurement error within the MR-Egger test.
CI = confidence interval. Df = Degrees of freedom.

Supplementary Table 10: SNPs excluded from the outlier corrected MR-PRESSO analyses between education and sedentary behaviours.

OutlierSNP	RSSobs	P-value
Education on television watching		
rs112634398	4.10E-04	0.0486
rs11712056	1.59E-04	<0.0486
rs148734725	1.19E-04	<0.0486
rs34072092	2.32E-04	<0.0486
Education on computer use		
rs11210860	7.21E-05	0.0486
rs13294439	2.00E-04	<0.0486
rs1402025	8.56E-05	0.0486
rs148734725	7.99E-05	<0.0486
rs17167170	1.06E-04	<0.0486
rs2431108	1.38E-04	<0.0486
rs34072092	2.32E-04	<0.0486
rs4863692	1.20E-04	<0.0486
rs9320913	1.16E-04	<0.0486
Education on driving		
rs2568955	7.78E-05	0.0486

Excluded variants are shown for the analyses between education and television watching, computer use and driving. MR-PRESSO does not return exact P-values in case variants are filtered and therefore non-exact P-values are shown.
OutlierSNP= Single nucleotide polymorphism removed from the analysis by MR-PRESSO. RSSobs= observed residual sum of squares. P-value cutoff = the cut-off value for the SNPs to be included in the Mendelian randomization analyses.

Supplementary Table 11: Q_{x1} and Q_{x2} and Q_a in the two-sample multivariable MR between sedentary behaviours, education and CAD.

Exposure	Q_{x1} and Q_{x2}				Q_a				P-value cut off
	Q_{x1}	Q_{x2}	df	Min Q_x	Q_a	df	Min Q_a	P-value	
Television	423.5	387.1	124	99.3	188.3	123	98.4	1.40E-04	1.00E-08
Television	638.8	566.4	177	147.2	261.1	176	146.3	3.26E-05	5.00E-08
Television	710.4	623.0	209	176.5	295.0	208	175.6	6.79E-05	1.00E-07
Television	1095.3	984.7	365	321.7	505.0	364	320.8	1.33E-06	1.00E-06
Television	2025.9	1704.3	704	643.4	950.5	703	642.5	1.17E-09	1.00E-05
Computer	173.7	139.5	30	18.5	46.0	29	17.7	2.37E-02	1.00E-08
Computer	229.2	181.6	45	30.6	74.1	44	29.8	3.05E-03	5.00E-08
Computer	283.1	232.3	54	38.1	91.6	53	37.3	7.90E-04	1.00E-07
Computer	654.7	444.3	110	86.8	146.3	109	85.9	9.99E-03	1.00E-06
Computer	2067.7	959.3	266	229.2	355.3	265	228.3	1.75E-04	1.00E-05
Driving	21.2	15.8	2	0.1	1.6	1	0.0	2.10E-01	1.00E-08
Driving	23.7	17.3	3	0.4	3.8	2	0.1	1.49E-01	5.00E-08
Driving	35.7	25.2	5	1.1	3.8	4	0.7	4.29E-01	1.00E-07
Driving	299.4	157.8	24	13.8	44.1	23	13.1	5.17E-03	1.00E-06
Driving	1415.8	349.3	93	71.8	96.6	92	70.9	3.51E-01	1.00E-05

Qx1 and Qx2 are measures of weak-instrument bias in a two-sample multivariable MR setting. Qa is a measure of heterogeneity. We estimated the critical value for the χ^2 (min Qx and min Qa) distribution using the amount of SNPs minus respectively two and three degrees of freedom at an one-sided P-value of 0.05.
Df = Degrees of freedom.

Supplementary Table 12: Results of the additional two-sample Mendelian randomization analyses between sedentary behaviours and cardiovascular risk factors, and subsequent multivariable Mendelian randomization analyses on CAD.

exposure2	outcome	method	nsnp	beta	se	*P-value*	OR	95% CI min	95% CI plus	*P-value* cut-off
Leisure television watching as primary exposure										
NA	BMI	IVW	144	-2.197	0.061	1.35E-282	NA	NA	NA	1.00E-08
BMI	CAD	MV-MR	127	0.245	0.099	1.30E-02	1.277	1.053	1.549	1.00E-08
NA	DM2	IVW	144	-0.838	0.072	5.22E-31	0.433	0.375	0.498	1.00E-08
DM2	CAD	MV-MR	127	0.270	0.086	1.64E-03	1.310	1.107	1.550	1.00E-08
NA	SBP	IVW	144	-2.767	0.209	5.44E-40	NA	NA	NA	1.00E-08
SBP	CAD	MV-MR	127	0.334	0.083	5.45E-05	1.397	1.188	1.643	1.00E-08
NA	DBP	IVW	144	-1.672	0.106	2.90E-56	NA	NA	NA	1.00E-08
DBP	CAD	MV-MR	127	0.343	0.085	5.60E-05	1.409	1.193	1.665	1.00E-08
NA	Hypertension	IVW	144	-0.620	0.048	7.52E-38	0.538	0.490	0.591	1.00E-08
Hypertension	CAD	MV-MR	127	0.225	0.085	8.21E-03	1.253	1.060	1.481	1.00E-08
NA	HDL	IVW	144	0.112	0.005	9.40E-119	NA	NA	NA	1.00E-08
HDL	CAD	MV-MR	127	0.260	0.094	5.79E-03	1.297	1.078	1.561	1.00E-08
NA	LDL	IVW	144	0.001	0.011	9.64E-01	NA	NA	NA	1.00E-08
LDL	CAD	MV-MR	127	0.365	0.073	6.20E-07	1.440	1.248	1.662	1.00E-08
NA	Triglyceride	IVW	144	-0.255	0.013	5.72E-83	NA	NA	NA	1.00E-08
Triglyceride	CAD	MV-MR	127	0.307	0.094	1.13E-03	1.359	1.130	1.635	1.00E-08
NA	TC	IVW	144	0.054	0.015	2.62E-04	NA	NA	NA	1.00E-08
TC	CAD	MV-MR	127	0.359	0.073	9.73E-07	1.432	1.240	1.654	1.00E-08
Leisure computer use as primary exposure										
NA	BMI	IVW	34	0.117	0.128	3.59E-01	NA	NA	NA	1.00E-08
BMI	CAD	MV-MR	32	-0.212	0.154	1.68E-01	0.809	0.598	1.093	1.00E-08
NA	DM2	IVW	34	-0.079	0.150	5.96E-01	0.924	0.689	1.239	1.00E-08
DM2	CAD	MV-MR	32	-0.213	0.158	1.78E-01	0.808	0.593	1.102	1.00E-08

NA	SBP	IVW	34	4.069	0.436	1.02E-20	NA	NA	NA	1.00E-08
SBP	CAD	MV-MR	32	-0.116	0.185	5.30E-01	0.890	0.620	1.280	1.00E-08
NA	DBP	IVW	34	1.430	0.221	8.93E-11	NA	NA	NA	1.00E-08
DBP	CAD	MV-MR	32	-0.199	0.169	2.40E-01	0.820	0.588	1.142	1.00E-08
NA	Hypertension	IVW	34	0.147	0.100	1.42E-01	1.158	0.952	1.409	1.00E-08
Hypertension	CAD	MV-MR	32	-0.194	0.153	2.03E-01	0.823	0.610	1.111	1.00E-08
NA	HDL	IVW	34	-0.010	0.010	3.10E-01	NA	NA	NA	1.00E-08
HDL	CAD	MV-MR	32	-0.216	0.155	1.65E-01	0.806	0.594	1.093	1.00E-08
NA	LDL	IVW	34	0.077	0.024	1.32E-03	NA	NA	NA	1.00E-08
LDL	CAD	MV-MR	32	-0.175	0.160	2.75E-01	0.840	0.613	1.150	1.00E-08
NA	Triglyceride	IVW	34	0.076	0.028	5.53E-03	NA	NA	NA	1.00E-08
Triglyceride	CAD	MV-MR	32	-0.246	0.156	1.15E-01	0.782	0.576	1.062	1.00E-08
NA	TC	IVW	34	0.104	0.031	8.12E-04	NA	NA	NA	1.00E-08
TC	CAD	MV-MR	32	-0.177	0.161	2.73E-01	0.838	0.611	1.149	1.00E-08
Driving as primary exposure										
NA	BMI	IVW	4	0.022	0.382	9.53E-01	NA	NA	NA	1.00E-08
BMI	CAD	MV-MR	4	0.962	0.194	6.69E-07	2.617	1.791	3.824	1.00E-08
NA	DM2	IVW	4	-0.677	0.449	1.32E-01	0.508	0.211	1.225	1.00E-08
DM2	CAD	MV-MR	4	0.840	0.289	3.58E-03	2.317	1.316	4.080	1.00E-08
NA	SBP	IVW	4	0.227	1.298	8.61E-01	NA	NA	NA	1.00E-08
SBP	CAD	MV-MR	4	0.981	0.337	3.61E-03	2.666	1.377	5.160	1.00E-08
NA	DBP	IVW	4	0.203	0.658	7.57E-01	NA	NA	NA	1.00E-08
DBP	CAD	MV-MR	4	0.964	0.336	4.11E-03	2.621	1.357	5.061	1.00E-08
NA	Hypertension	IVW	4	0.110	0.299	7.14E-01	1.116	0.621	2.006	1.00E-08
Hypertension	CAD	MV-MR	4	1.000	0.340	3.25E-03	2.720	1.397	5.295	1.00E-08
NA	HDL	IVW	4	0.222	0.030	1.39E-13	NA	NA	NA	1.00E-08
HDL	CAD	MV-MR	4	0.719	0.077	6.06E-21	2.053	1.767	2.385	1.00E-08
NA	LDL	IVW	4	-0.233	0.071	1.09E-03	NA	NA	NA	1.00E-08
LDL	CAD	MV-MR	4	0.826	0.317	9.12E-03	2.283	1.228	4.248	1.00E-08
NA	Triglyceride	IVW	4	-0.447	0.082	5.58E-08	NA	NA	NA	1.00E-08

Triglyceride	CAD	MV-MR	4	0.776	0.231	8.02E-04	2.172	1.380	3.419	1.00E-08
NA	TC	IVW	4	-0.122	0.092	1.86E-01	NA	NA	NA	1.00E-08
TC	CAD	MV-MR	4	0.953	0.360	8.06E-03	2.593	1.281	5.248	1.00E-08

Traditional cardiovascular risk factors included body mass index, history of diabetes, systolic blood pressure, diastolic blood pressure, history of hypertension and lipid profile. First, MR analyses between sedentary behaviours and the secondary phenotype were performed. Next, a multivariable MR between sedentary behaviours, the secondary trait and CAD was performed. Only variants at $P < 1 \times 10^{-8}$ *were used. IVW stands for inverse variance weighted (in this case, fixed effects models were performed), MV-MR stands for multivariable Mendelian randomization.*
BMI stands for body mass index, DM2 for a history of diabetes mellitus type 2, SBP for systolic blood pressure, DBP for diastolic blood pressure, hypertension for a medical history of hypertension, HDL for high density lipoprotein, LDL for low densitiy lipoprotein and TC for total cholesterol. NA stands for not applicable, and is provided in case 1) there was no secondary exposure 2) the outcome was a linear trait and odds ratios could not be provided.
Exposure2 = secondary exposure, nsps = number of snps, OR = odd ratio, CI = confidence interval.

Supplementary Discussion

Several sensitivity MR analyses were performed to investigate the causal association between sedentary behaviours and CAD, with each of these tests harboring their own strengths and weaknesses. First of all, it is important that genetic variants do explain enough variance of its original trait. We therefore assessed weak instruments bias by examining the F-statistics, which were above 10 for all genetic variants indicating that current analyses do not suffer from weak instrument bias. I^2_{GX} indicated that the MR-Egger estimates of driving may have suffered from weak instrument bias in the main analysis and these results should therefore be interpreted with caution.

Although weak instrument bias was carefully assessed, our analyses do not provide evidence for the specificity of the discovered genetic instruments for its phenotype. The genetic variants could still exert an effect on CAD through generic pathways or correlated traits as education, i.e. pleiotropy. Indeed it would be remarkable if a neurologically driven trait as sedentary behaviours, shown to be correlated with other neurologically driven traits, would not influence these traits or vice versa. Regular inverse variance weighted MR analyses should in such a scenario be interpreted with caution, as this would violate its' assumptions. We believe such pleiotropy to be unlikely for several reasons.

First of all, we assessed heterogeneity and thus potential horizontal pleiotropy using the Rucker framework[1] and found evidence for balanced pleiotropy in the association between television watching and CAD. In this scenario, a causal effect is properly estimated using an inverse variance weighted random effects model. The causal estimate between television watching and CAD remained similar to the main analyses, only with slightly broader confidence intervals. To further explore horizontal pleiotropy, we performed more stringent analyses using the latest MR methods, including MR-Egger[2], exclusion of potentially pleiotropic variants in MR-IVW analysis and MR-PRESSO[3].

Secondly, we assessed whether pleiotropy was due to horizontal or vertical pleiotropy using multiple multivariable MR in which we corrected for correlated traits including education or other cardiovascular risk factors[4]. This test has its weaknesses (i.e. potential bias in the case of overlapping risk factors and potential to suffer from weak instrument bias[5]), but also strengths (i.e. control for potential pleiotropy due to correlated traits including education or other cardiovascular risk factors). The multivariable MR in which we corrected for education showed a similar effect as the main analyses, although the confidence intervals were broader. Although the genetics underlying television watching and education are clearly intertwined (as shown by the high genetic correlations), there seems to be an independent effect of television watching on CAD in our estimates. Q_{x1} and Q_{x2} indicated that all primary exposures (sedentary behaviours) and the secondary exposure (education) did not suffer from weak instrument bias in any of the analyses. Q_a indicated heterogeneity and thus potential pleiotropy in the associations between television watching (and computer use) with CAD when already corrected for education. This indicates that we cannot rule out that other traits are on the causal pathway between television watching and CAD, whether it is due to vertical pleiotropy (as discussed below) or horizontal pleiotropy due to currently uninvestigated traits. Based on the fact that only neurological pathways are highlighted for television watching, it would be surprising to see that television watching immediately increases risk of CAD. We therefore performed additional multivariable MR analyses, proven to be a proper approach for mediation analyses[6], to investigate whether traditional cardiovascular risk factors were on the causal pathway to CAD. Indeed, we did observe vertical pleiotropy when correcting for these risk factors, as the total effect of television watching on CAD was attenuated compared to the direct effect. In case the association between television watching and CAD is true, this is likely mediated by common cardiovascular risk factors as BMI, lipid profile and blood pressure, in line with results from previous observational studies[7–10]. It is important to note that the attenuation of the effect in the multivariate MR corrected for traditional cardiovascular risk factors is possibly accentuated by assessing the secondary exposures' effect estimates within the same cohort. The multivariable MR's do not prove uniqueness of the instruments used in the MR analyses. If any, it further supports overlap with neurologically driven traits as education and BMI. Our results indicate horizontal pleiotropy with education and vertical pleiotropy with common cardiovascular risk factors. However, they do provide evidence for independence of the association for education and possible pathways in which television watching exerts its effect on CAD.

Thirdly, pleiotropy through general (for example, mitochondrial function) or purely cardiovascular pathways (for example, coagulation) is not likely, as all the genetic variants for television watching showed enrichment for pathways involved in neurological development and were mainly enriched in neural tissue. Any pleiotropy is therefore most likely to be caused by neurological traits (for example, education or obesity inducing behaviours). We therefore performed rigorous sensitivity analyses to assess pleiotropy through these traits, as described above.

Lastly, we obtained evidence for an association for an association between television watching and CAD through multiple approaches, i.e. triangulation of evidence[11]. This is essential as different approaches harbor different

strengths and weaknesses. For example, observational studies are often hampered by confounding and reverse causation, which MR studies overcome. On the other hand, MR studies could potentially be affected by weak instrument bias and pleiotropy. The evidence for a possible causal relationship (as found in the MR) between television watching and CAD is therefore strengthened by congruent results with previous research[12] and current observational analyses.

In short, both observational and all MR analyses were consistent and therefore pointed to a causal interpretation of the association between television watching and CAD. We emphasize that no definitive tests exists to verify true causality and we stress careful interpretation of the results in the light of the potential complicated relationship between education and disease[13].

Supplementary References

1. Bowden, J. *et al.* A framework for the investigation of pleiotropy in two-sample summary data Mendelian randomization. *Stat. Med.* **36**, 1783–1802 (2017).
2. Bowden, J., Davey Smith, G. & Burgess, S. Mendelian randomization with invalid instruments: effect estimation and bias detection through Egger regression. *Int. J. Epidemiol.* **44**, 512–525 (2015).
3. Verbanck, M., Chen, C.-Y., Neale, B. & Do, R. Detection of widespread horizontal pleiotropy in causal relationships inferred from Mendelian randomization between complex traits and diseases. *Nat. Genet.* **50**, 693–698 (2018).
4. Sanderson, E., Davey Smith, G., Windmeijer, F. & Bowden, J. An examination of multivariable Mendelian randomization in the single-sample and two-sample summary data settings. *Int. J. Epidemiol.* (2018). doi:10.1093/ije/dyy262
5. Holmes, M. V & Davey Smith, G. Challenges in Interpreting Multivariable Mendelian Randomization: Might "Good Cholesterol" Be Good After All? *Am. J. Kidney Dis.* **71**, 149–153 (2018).
6. Carter, A. R. *et al.* Mendelian randomisation for mediation analysis: current methods and challenges for implementation. *bioRxiv* 835819 (2019). doi:10.1101/835819
7. Benatti, F. B. & Ried-Larsen, M. The Effects of Breaking up Prolonged Sitting Time: A Review of Experimental Studies. *Med. Sci. Sports Exerc.* **47**, 2053–2061 (2015).
8. Ainsworth, B. E. *et al.* 2011 compendium of physical activities: A second update of codes and MET values. *Med. Sci. Sports Exerc.* **43**, 1575–1581 (2011).
9. Frydenlund, G., Jørgensen, T., Toft, U., Pisinger, C. & Aadahl, M. Sedentary leisure time behavior, snacking habits and cardiovascular biomarkers: The Inter99 Study. *Eur. J. Prev. Cardiol.* **19**, 1111–1119 (2012).
10. Altenburg, T. M., de Kroon, M. L. A., Renders, C. M., HiraSing, R. & Chinapaw, M. J. M. TV Time but Not Computer Time Is Associated with Cardiometabolic Risk in Dutch Young Adults. *PLoS One* **8**, e57749 (2013).
11. Lawlor, D. A., Tilling, K. & Smith, G. D. Triangulation in aetiological epidemiology. *Int. J. Epidemiol.* **45**, 1866–1886 (2016).
12. Wijndaele, K. *et al.* Television Viewing and Incident Cardiovascular Disease: Prospective Associations and Mediation Analysis in the EPIC Norfolk Study. *PLoS One* **6**, e20058 (2011).
13. Davies, N. M. *et al.* Multivariable two-sample Mendelian randomization estimates of the effects of intelligence and education on health. *Elife* **8**, (2019).
