# Supplementary material for: Genome-wide association studies and Mendelian randomization analyses for leisure sedentary behaviours
Source: Nat Commun. 2020 Apr 21;11:1770. doi: 10.1038/s41467-020-15553-w (PMC7174427; doi:10.1038/s41467-020-15553-w)
Supplement: Supplementary file 17 — Description of Additional Supplementary Files [file 41467_2020_15553_MOESM17_ESM.pdf]

Title: Supplementary Data 1:
Description: 193 Novel genome-wide sedentary behaviour SNPs (.xlsx).

Title: Supplementary Data 2:
Description: Genetic correlation between sedentary behaviours, and previously performed GWAS's (.xlsx).

Title: Supplementary Data 3:
Description: Sedentary behaviour variants associated with previously discovered variants (.xlsx).

Title: Supplementary Data 4:
Description: List of coding variants (.xlsx).

Title: Supplementary Data 5:
Description: List of functional eQTL genes (.xlsx).

Title: Supplementary Data 6:
Description: List of DEPICT genes (.xlsx).

Title: Supplementary Data 7:
Description: Results of gene sets discovered by DEPICT for sedentary behaviour and sedentary behaviour traits separately (.xlsx).

Title: Supplementary Data 8:
Description: Results of enriched tissue sets discovered by DEPICT for sedentary behaviour and sedentary behaviour traits separately (.xlsx).

Title: Supplementary Data 9:
Description: Genetic association estimates for the association between sedentary behaviours and coronary artery disease (.xlsx).

Title: Supplementary Data 10:
Description: F-statistics and R2 of all instruments for the Mendelian randomization analysis between sedentary behaviours and coronary artery disease (.xlsx).

Title: Supplementary Data 11:
Description: Inverse variance weighted Mendelian randomization estimates for the association between education and sedentary behaviours (.xlsx).

Title: Supplementary Data 12:
Description: Genetic association estimates for the two-sample regression-based multivariable Mendelian randomization analyses estimating the effect of sedentary behaviours on coronary artery disease, adjusted for education (.xlsx).

Title: Supplementary Data 13:
Description: Genetic association estimates for the two-sample regression-based multivariable Mendelian randomization analyses estimating the effect of sedentary behaviours on coronary artery disease, adjusted for cardiovascular risk factors. (.xlsx).
